# Development and eruption of human teeth in the Chinese population: a comprehensive dental atlas

**DOI:** 10.3389/fdmed.2024.1434417

**Published:** 2024-08-27

**Authors:** Jayakumar Jayaraman

**Affiliations:** Department of Pediatric Dentistry, Virginia Commonwealth University School of Dentistry, Richmond, VA, United States

**Keywords:** dental atlas, Chinese, dental development, dental chart, primary teeth, permanent teeth, human dentition, forensics age estimation

## Abstract

**Aim:**

The first comprehensive chart on dental development was published 75 years ago based on Caucasian children and this has been used as a standard dental chart to date. Few population specific charts have been developed recently and updated dental charts on modern subjects can provide more information on dental development patterns. This study aims to construct a comprehensive dental atlas for modern Chinese children and young adults to assist in clinical, forensic, and public health applications.

**Methods:**

The study sample comprised of 2,306 subjects, age ranging from 2 to 24 years belonging to Chinese ethnicity. Dental formation and eruption of permanent teeth and resorption of primary teeth were analyzed separately for females and males. For each age range, the number of teeth (n), and the stage of development was calculated for first (Q1), second (Q2) and third quartiles (Q3). Similar analysis was performed for the position of permanent teeth and the resorption of primary teeth. To determine the variations between the sex, Mann-Whitney *U*-test was conducted by comparing the median (Q2) stages.

**Results:**

Variations in dental formation and eruption of permanent teeth and resorption of primary teeth were observed between maxillary and mandibular dentitions and between the sex, however the difference was not statistically significant (*p* = 0.535 to *p* = 1.000). The dental atlas was presented separately for Chinese females and males.

**Conclusion:**

This atlas on modern Chinese population serves as a practical tool to assist in clinical diagnosis and treatment planning, in forensic investigations as well as indicators of developments in public health.

## 1 Introduction

Dental development is a sequential process that passes through several stages of formation to erupt to its final position in the arch. The primary teeth and first permanent molars start to develop in-utero and skeletal specimens serve as an ideal tool to evaluate early stages of dental development. This is usually conducted by direct vision of the skeletal remains; however, radiological and histological evaluation can also be of some assistance. For permanent teeth, radiographs provide an excellent platform to record dental development. Different methods of staging dental development have been proposed in the literature that varies between 4 and 32 stages (1–3); the most accepted being the eight-stage Anglo-Canadian classification system (1). Similarly, the stages of resorption of roots of primary teeth have been analyzed that classified into 4 and 5 stages respectively (3, 4). The eruption of teeth has been evaluated and a five-stage classification system was proposed to relate the position of the tooth in the arch (5). This original system has been modified into a four-stage system in the London Atlas study (4).

The first comprehensive chart on dental development was published in 1941 based on Caucasian children in the United States and this has been commonly used as a standard dental atlas chart for several decades (6). The reliability of using this chart has been questioned due to the smaller number of samples in each age group. This chart was based on histological specimens of only 25 children out of which only seven were in the age range of 2 to 15 years (7). Several investigators have shown the presence of population differences in dental development (8, 9). Considering this, population specific charts has been developed and this includes London Atlas based on Caucasian and Bangladeshi populations in London (4), Australian Atlas (10, 11), and most recently, WITS Atlas based on southern African population (12).

Chinese constitute one of the major population and occupies one fifth of the total human population in the world and about 94% of people living in Hong Kong are of Chinese, predominantly of Cantonese origin but there was some admixture of other subgroups (13). The data on eruption of permanent teeth in Chinese was first presented 50 years ago and was not updated since then (14). Moreover, their study did not analyze the entire picture of dental development namely dental formation, eruption and resorption. To our understanding, there was no comprehensive dental atlas for Chinese population. This study was aimed to evaluate dental development pattern and subsequently construct dental atlas for Chinese children and young adults.

## 2 Materials and methods

### 2.1 Study population

The study comprised of 2,306 subjects, age ranging from 2 to 24 years with equal number of males and females, refer to Table 1. All the subjects were of southern Chinese ethnicity belonging to middle socioeconomic status and the Dental Panoramic Tomographs (DPT) were obtained from the archives of a teaching hospital in Hong Kong, Special Administrative Region of China. The radiographs were previously scored for a Dental Age Estimation study and have been re-used in the current study (15). The study was approved by the Institutional Review Board (Reference No: UW 12-280). The author retains ownership of the collected dataset and subsequent secondary analysis of the de-identified data presented in this manuscript. Only healthy subjects are included and those with severe anomalies that might affect dental development were excluded from the study. All the radiographs were scored by a single trained and calibrated examiner (JJ) with intra-examiner reliability Kappa score of 0.85. These scores correspond to “almost perfect” correlation (16). The radiographs were digitized at 300 dpi in gray scale format using a flatbed scanner and viewed in a dark room on a wide screen monitor at a magnification rate of 160% (Philips 271E, Philips Industries, Taiwan). When in doubt, the digitized images were enlarged up to 300% for better evaluation. Each tooth was designated by an alphabet followed by a number, for example, UL1 refers to Upper Left Central Incisor, UL2 refers to Upper Left Lateral Incisor and so on. The median (Q2), lower (Q1) and upper (Q3) interquartile ranges for each tooth was calculated separately for males and females. This analysis was conducted for formation and eruption of permanent dentition and resorption of primary dentition at different age ranges. The median stages are compared in this study due to the assumption that the data is not normally distributed. To determine the variations in dental development in males and females, Mann-Whitney *U*-test was conducted by comparing the median (Q2) stages of dental formation, eruption stages and resorption stages (SPSS Version 20.0, IBM Corp, Armonk, NY).

**Table 1:** Distribution of Chinese subjects used to analyse the formation and eruption of permanent teeth and resorption of primary teeth.

	Maturation^b^		Eruption^b^		Resorption^c^	
Age^a^	Males	Females	Males	Females	Males	Females
2.5	52	53	20	20	20	20
3.5	46	50	20	20	20	20
4.5	50	56	20	20	20	20
5.5	98	99	20	20	20	20
6.5	58	52	20	20	20	20
7.5	50	55	20	20	20	20
8.5	49	50	20	20	20	20
9.5	49	50	20	20	20	20
10.5	48	50	20	20	20	20
11.5	57	44	20	20	20	20
12.5	47	49	20	20	20	20
13.5	51	54	20	20	20	20
14.5	53	49	20	20	20	20
15.5	44	43	20	20	–	–
16.5	42	45	20	20	–	–
17.5	47	56	20	20	–	–
18.5	70	36	20	20	–	–
19.5	32	33	20	20	–	–
20.5	51	43	20	20	–	–
21.5	54	37	20	20	–	–
22.5	40	38	20	20	–	–
23.5	43	37	20	20	–	–
24.5	52	44	20	20	–	–
**Total**	**1,183**	**1,123**	**460**	**460**	**260**	**260**

^a^ Age in midpoint of 1 year.

^b^ Permanent dentition.

^c^ Primary dentition.

–No data available as the tooth underwent normal physiological exfoliation.

### 2.2 Formation of permanent teeth

**Figure 1:**
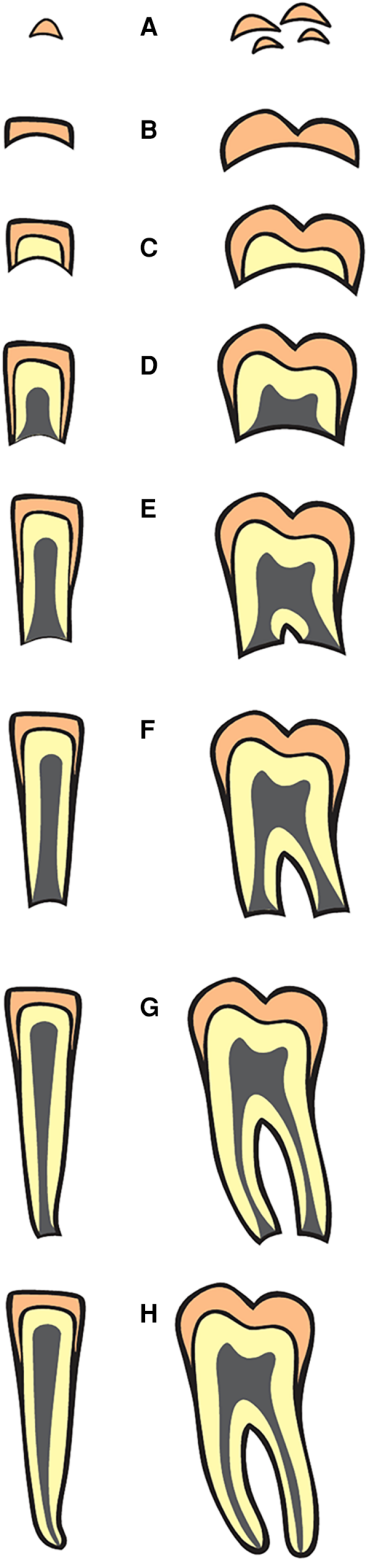
Classification of staging of dental formation (Stages A to H) in single and multi-rooted teeth (Data source: reference (1)).

Formation of permanent tooth was evaluated from 2,306 subjects based on Anglo-Canadian standards of dental formation designated in alphabets stages A to H, starting with initial calcification of tooth (stage A) up to completion of the root development (stage H), see Figure 1. All the teeth on the left side of the arch were evaluated for the stage of development (1). When a tooth in the left side is missing, the corresponding tooth on the right side was scored. The stages were recorded on a score card including the details of sex, ethnicity, date of birth and the date of exposure of the radiograph. The data was entered in the Microsoft Access database to calculate the average age for each corresponding stage of development (TDS) for each tooth morphology type (TMT). The details were then transferred to Microsoft Excel spreadsheet separately for males and females aged 2 to 24 years. For each age range, the number of teeth (n) and the corresponding stage of development were calculated for lower quartile (Q1), second quartile (Q2) and upper quartile (Q3) ranges. The stage corresponding to median (Q2) was used to develop dental formation charts for each age range, and separately for males and females.

### 2.3 Eruption of permanent teeth

**Figure 2:**
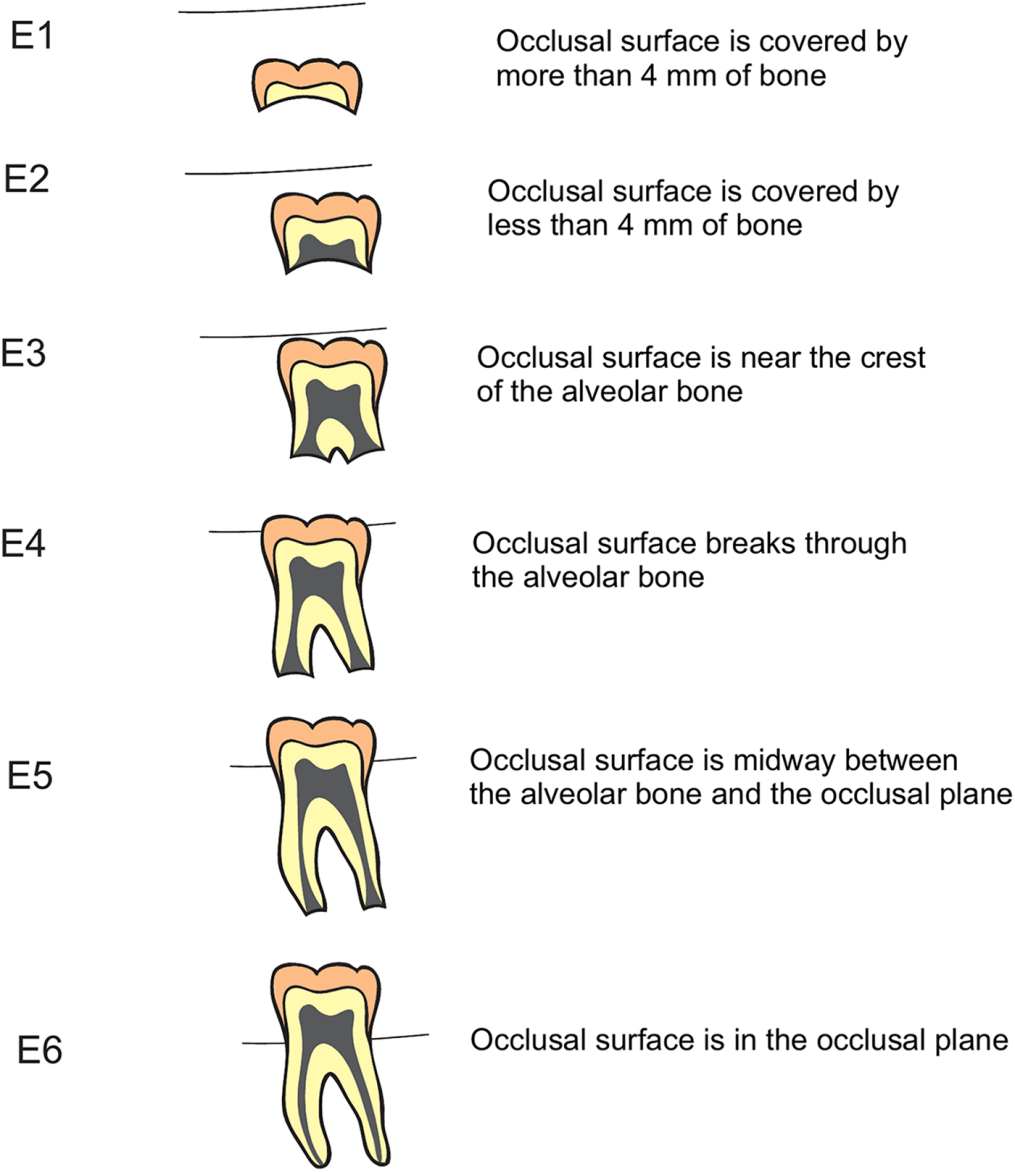
Classification of staging of eruption of teeth (Stages E1 to E6) in relation to alveolar bone level (Data source modified from: reference (5)).

To evaluate the position of permanent teeth in the arch, a total of 920 radiographs were randomly chosen from the total dataset partitioned by age and sex. This comprised twenty radiographs per age and sex of subjects aged 2 to 24 years. The most used classification stages of eruption of teeth follows five stages designated by P-I to P-V. Position P-I corresponds to tooth in full occlusion and P-V signifies the position of the tooth below the apical third of the primary root or more than 2 mm below the bone when the predecessor is absent. This observation was based on the permanent molars (5). However, this classification system did not consider of position of permanent teeth at subsequent categorical stages of development. In this study, a new classification system for staging dental eruption was adopted designated as E1 to E6, where E1 to E3 infers intra-osseous and E4 to E6 corresponds to extra-osseous positioning of permanent teeth. Stage E4 refers to radiographic position of tooth just above the alveolar bone level. A detailed description of this classification system was presented in Figure 2.

### 2.4 Resorption of primary teeth

**Figure 3:**
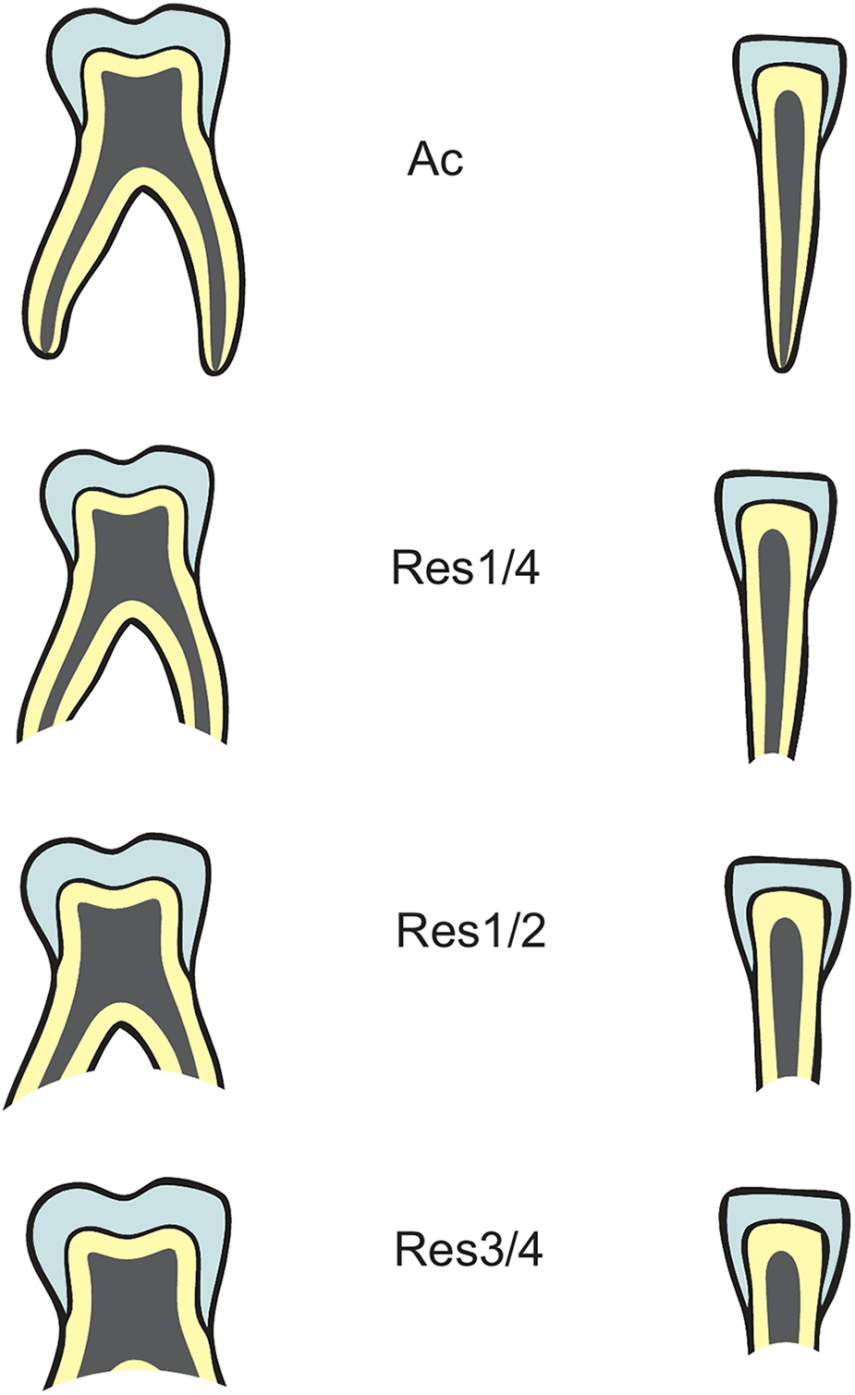
Classification of resorption patterns (Stages Ac, Res1/4, Res1/2, Res3/4) of single and multi-rooted primary teeth (Data source: reference (3)).

A total of 520 radiographs, comprising of 20 radiographs for each sex and age between ages 2 and 14 were chosen to evaluate the resorption pattern of primary teeth (3). This sample was randomly obtained from the same data used to analyze eruption pattern of permanent teeth. Resorption of maxillary and mandibular primary teeth was analyzed by stages defined by Moorrees and co-workers; Ac corresponds to tooth with complete root development without radiological signs of resorption, Res1/4 showing one quarter root resorption, Res1/2 and Res3/4 corresponding to half and three quarters of resorption respectively, see Figure 3.

## 3 Results

### 3.1 Assessment of dental development

Mann-Whitney *U*-test was used to compare the median stage (Q2) of formation between males and females in the maxillary and mandibular dentitions at each age range from 2 to 24 years. One or two stage difference was observed in the median stages of dental formation of permanent teeth between maxillary and mandibular dentitions and between the sex. Between sexes, stage difference in formation of teeth was observed in all the age ranges except 3 and 7 years in the maxillary dentition and 4, 7, and 14 years in the mandibular dentition. The difference was not statistically significant between the sexes across all the age ranges (*p* = 0.535 to *p* = 1.000), see Table 2.

**Table 2:** Formation of maxillary and mandibular permanent teeth of 2 to 24 years old Chinese females and males.

MAXILLA											MANDIBLE									
Age^b^	Teeth^c^	Males				Females					Teeth^c^	Males				Females				
		*n*	Q1	Q2	Q3	*n*	QI	Q2	Q3	*p*-value		*n*	Q1	Q2	Q3	*n*	QI	Q2	Q3	*p*
2.5	ULI	46	D	D	D	41	D	D	D		LL1	49	D	D	D	49	D	D	D	
	UL2	40	C	C	D	38	B	B	D		LL2	49	C	D	D	49	C	C	D	
	UL3	38	C	C	C	44	B	B	B		LL3	50	C	C	C	51	C	C	C	
	UL4	^a^	^a^	^a^	^a^	^a^	^a^	^a^	^a^		LL4	29	A	A	B	26	A	A	B	
	UL5	^a^	^a^	^a^	^a^	^a^	^a^	^a^	^a^		LL5	^a^	^a^	^a^	^a^	^a^	^a^	^a^	^a^	
	UL6	49	D	D	D	40	D	D	D	0.710	LL6	42	D	D	D	37	D	D	D	0.805
3.5	ULI	44	D	D	D	50	D	D	D		LL1	44	D	D	E	50	D	D	E	
	UL2	43	C	D	D	49	D	D	D		LL2	40	D	D	D	50	D	D	D	
	UL3	44	C	D	D	49	B	D	D		LL3	45	C	D	D	50	C	C	D	
	UL4	38	B	B	B	44	B	B	B		LL4	44	B	B	C	49	B	B	C	
	UL5	18	A	B	B	13	A	B	B		LL5	30	A	B	B	28	A	A	B	
	UL6	46	D	D	D	49	D	D	D		LL6	46	D	D	D	50	D	D	D	
	UL7	19	A	A	B	16	B	B	B	1.000	LL7	27	A	A	B	22	A	A	A	0.710
4.5	ULI	50	D	D	D	56	D	D	D		LL1	50	D	E	E	56	D	E	E	
	UL2	49	D	D	D	56	D	D	D		LL2	50	D	D	D	56	D	D	D	
	UL3	48	D	D	D	56	D	D	D		LL3	50	D	D	D	56	D	D	D	
	UL4	50	B	B	C	55	B	C	C		LL4	50	B	C	C	56	B	C	C	
	UL5	43	A	B	B	45	B	B	B		LL5	45	B	B	B	52	B	B	B	
	UL6	50	D	D	E	56	D	D	E		LL6	50	D	E	E	56	D	E	E	
	UL7	46	A	B	B	43	B	B	B	0.902	LL7	29	B	B	B	31	B	B	B	1.000
5.5	ULI	96	D	E	E	98	E	E	E		LL1	99	E	F	F	95	E	F	F	
	UL2	97	D	D	E	97	D	E	E		LL2	95	E	E	E	97	E	E	E	
	UL3	99	D	D	D	98	D	D	E		LL3	99	D	D	D	99	D	D	E	
	UL4	100	C	D	D	98	D	D	D		LL4	99	C	D	D	99	D	D	D	
	UL5	92	C	C	D	93	C	C	D		LL5	99	C	C	D	98	C	C	D	
	UL6	99	E	E	F	99	E	F	F		LL6	100	E	E	F	98	E	F	F	
	UL7	98	C	C	C	98	C	C	D	0.902	LL7	97	B	C	C	98	C	C	C	0.902
6.5	ULI	58	E	F	F	52	E	E	F		LL1	58	F	F	F	52	F	F	F	
	UL2	58	D	E	E	51	E	E	E		LL2	56	E	E	F	52	E	F	F	
	UL3	58	D	D	E	51	D	E	E		LL3	58	D	D	E	51	D	E	E	
	UL4	57	D	D	D	52	D	D	D		LL4	58	D	D	D	52	D	D	D	
	UL5	58	C	D	D	50	C	D	D		LL5	56	C	D	D	50	C	D	D	
	UL6	55	F	F	F	52	F	F	G		LL6	55	F	F	F	52	F	F	G	
	UL7	58	C	D	D	50	C	D	D	0.902	LL7	57	C	C	D	51	C	D	D	0.535
7.5	ULI	48	F	F	F	55	F	F	F		LL1	50	G	G	G	55	F	G	G	
	UL2	48	E	F	F	53	E	F	F		LL2	49	F	G	G	55	F	F	G	
	UL3	49	E	E	E	55	E	E	F		LL3	49	D	E	E	55	E	E	F	
	UL4	49	D	D	D	54	D	D	E		LL4	49	D	D	E	54	D	E	E	
	UL5	49	D	D	E	54	D	D	D		LL5	49	D	D	D	54	D	D	E	
	UL6	49	G	G	G	55	G	G	G		LL6	50	G	G	G	54	G	G	G	
	UL7	49	D	D	D	55	D	D	D	1.000	LL7	50	D	D	D	55	D	D	D	1.000
8.5	ULI	48	F	F	G	50	F	G	G		LL1	48	G	H	H	49	H	H	H	
	UL2	48	F	F	F	49	F	F	G		LL2	49	F	G	G	50	G	G	H	
	UL3	49	E	E	F	48	E	F	F		LL3	48	E	E	F	49	E	F	F	
	UL4	48	D	E	E	47	D	D	E		LL4	49	E	E	E	48	E	E	E	
	UL5	48	D	D	E	48	D	E	E		LL5	49	D	E	E	50	D	E	E	
	UL6	48	G	H	H	49	H	H	H		LL6	49	G	G	H	50	H	H	H	
	UL7	48	D	D	D	50	D	D	D	0.721	LL7	49	D	D	D	50	D	D	D	0.798
9.5	ULI	46	F	G	G	48	G	G	H		LL1	48	H	H	H	28	H	H	H	
	UL2	42	F	G	G	47	F	G	G		LL2	48	G	H	H	48	G	H	H	
	UL3	46	F	F	F	49	F	F	F		LL3	48	F	F	F	48	F	F	G	
	UL4	49	E	F	F	49	E	F	F		LL4	49	E	F	F	50	F	F	F	
	UL5	49	E	E	F	50	E	F	F		LL5	47	E	E	F	49	E	F	F	
	UL6	49	H	H	H	50	H	H	H		LL6	–	–	–	–	–	–	–	–	
	UL7	49	D	D	E	50	D	D	E		LL7	49	D	D	E	50	D	E	E	
	UL8	21	A	B	B	23	A	B	B	0.382	LL8	25	A	A	B	25	A	A	B	0.798
10.5	ULI	45	G	H	H	47	H	H	H		LL1	26	H	H	H	26	H	H	H	
	UL2	45	G	H	H	47	G	H	H		LL2	46	H	H	H	26	G	H	H	
	UL3	47	F	F	F	44	F	G	G		LL3	47	F	F	F	49	F	G	G	
	UL4	44	F	F	F	45	F	F	G		LL4	48	F	F	F	50	F	F	G	
	UL5	44	F	F	F	43	F	F	G		LL5	48	F	F	F	50	F	F	F	
	UL6	–	–	–	–	–	–	–	–		LL6	–	–	–	–	–	–	–	–	
	UL7	48	D	E	E	49	E	E	F		LL7	48	E	E	E	50	E	E	F	
	UL8	30	A	B	B	30	B	B	C	0.442	LL8	35	A	A	B	35	A	B	C	0.645
11.5	ULI	–	–	–	–	–	–	–	–		LL1	–	–	–	–	–	–	–	–	
	UL2	–	–	–	–	–	–	–	–		LL2	–	–	–	–	–	–	–	–	
	UL3	52	F	F	G	42	G	G	H		LL3	56	F	F	G	42	G	G	H	
	UL4	50	F	G	G	38	F	G	H		LL4	56	F	G	G	43	F	G	H	
	UL5	54	F	F	G	38	F	G	G		LL5	55	F	F	G	43	F	F	G	
	UL6	–	–	–	–	–	–	–	–		LL6	–	–	–	–	–	–	–	–	
	UL7	56	E	F	G	43	E	F	G		LL7	56	E	F	G	40	E	F	G	
	UL8	37	B	C	C	34	B	C	D	0.382	LL8	44	B	C	C	35	B	C	D	0.645
12.5	ULI	–	–	–	–	–	–	–	–		LL1	–	–	–	–	–	–	–	–	
	UL2	–	–	–	–	–	–	–	–		LL2	–	–	–	–	–	–	–	–	
	UL3	41	F	G	G	48	G	G	H		LL3	47	F	G	H	47	G	G	H	
	UL4	38	G	G	H	48	G	H	H		LL4	47	G	G	H	48	G	G	H	
	UL5	43	G	G	G	49	G	G	H		LL5	46	F	G	G	49	F	G	G	
	UL6	–	–	–	–	–	–	–	–		LL6	–	–	–	–	–	–	–	–	
	UL7	46	F	G	G	48	F	G	G		LL7	46	F	G	G	49	F	G	G	
	UL8	35	C	D	D	40	C	D	D	0.721	LL8	42	C	D	D	42	C	C	D	0.574
13.5	ULI	–	–	–	–	–	–	–	–		LL1	–	–	–	–	–	–	–	–	
	UL2	–	–	–	–	–	–	–	–		LL2	–	–	–	–	–	–	–	–	
	UL3	49	G	G	H	52	G	H	H		LL3	33	G	G	H	54	H	H	H	
	UL4	49	G	H	H	50	H	H	H		LL4	50	G	H	H	53	H	H	H	
	UL5	50	G	H	H	51	G	H	H		LL5	50	G	G	H	52	G	H	H	
	UL6	–	–	–	–	–	–	–	–		LL6	–	–	–	–	–	–	–	–	
	UL7	50	G	G	H	52	G	G	H		LL7	49	G	G	H	54	G	G	H	
	UL8	38	D	D	D	48	D	D	D	0.878	LL8	45	D	D	D	46	D	D	D	0.721
14.5	ULI	–	–	–	–	–	–	–	–		LL1	–	–	–	–	–	–	–	–	
	UL2	–	–	–	–	–	–	–	–		LL2	–	–	–	–	–	–	–	–	
	UL3	52	H	H	H	43	G	H	H		LL3	16	G	G	G	12	G	G	H	
	UL4	16	H	H	H	10	G	G	H		LL4	20	G	H	H	22	G	H	H	
	UL5	26	G	H	H	15	G	G	H		LL5	53	H	H	H	49	H	H	H	
	UL6	–	–	–	–	–	–	–	–		LL6	–	–	–	–	–	–	–	–	
	UL7	52	G	H	H	49	G	H	H		LL7	53	G	H	H	49	G	H	H	
	UL8	44	D	D	D	44	D	D	D	0.721	LL8	49	D	D	E	47	D	D	E	1.000
15.5	UL8	34	D	D	E	33	D	D	E		LL8	39	D	E	F	40	D	D	E	
16.5	UL8	38	D	E	G	42	D	E	F		LL8	39	D	E	F	43	D	E	F	
17.5	UL8	36	E	F	G	44	D	E	F		LL8	43	F	F	G	51	E	F	G	
18.5	UL8	54	F	G	G	27	E	F	G		LL8	59	G	G	G	33	F	G	G	
19.5	UL8	26	G	H	H	25	F	G	H		LL8	29	G	G	G	25	G	G	G	
20.5	UL8	34	G	H	H	33	F	G	H		LL8	44	G	G	H	34	G	G	H	
21.5	UL8	41	G	H	H	31	G	H	H		LL8	43	G	H	H	27	G	H	H	
22.5	UL8	32	H	H	H	25	H	H	H		LL8	38	G	H	H	34	H	H	H	
23.5	UL8	37	H	H	H	28	H	H	H		LL8	39	H	H	H	32	H	H	H	
24.5	UL8	40	H	H	H	30	H	H	H	0.529	LL8	46	H	H	H	38	H	H	H	0.971

Q1- lower quartile, Q2- median, Q3- upper quartile.

n - Number of teeth, *p* - Level of significance.

^a^ No data available to assess the stage of dental development.

^b^ Age in midpoint of one year.

^c^ Tooth nomenclature adopted from the British Dental Journal nomenclature system.

–Indicates Stage H (complete root development) and the corresponding data not included in the analysis.

The median positioning of the permanent teeth as indicated by the rate of eruption varied between males and females and between the dentitions. In both maxillary dentition, and mandibular dentitions, difference in eruption pattern between the sexes was observed more in the younger age ranges and the most varying presentation in the eruption was observed in the third molars in both maxillary dentitions. Mann-Whitney *U*-test no statistically significant difference between the sexes in all the age groups (*p* = 0.721 to *p* = 1.000), see Table 3.

**Table 3:** Position of maxillary and mandibular permanent teeth in the arches of 2 to 21 years old Chinese females and males.

Maxilla										
Age*	Gender	UL1	UL2	UL3	UL4	UL5	UL6	UL7	UL8	*p*-value^^^
2.5	♂	1	2	1	1	–	2	–	–	
	♀	2	2	1	1	–	3	–	–	0.721
3.5	♂	1	2	1	1	1	3	1	–	
	♀	2	2	1	1	1	3	1	–	0.798
4.5	♂	2	2	1	1	1	3	1	–	
	♀	2	2	1	1	1	3	1	–	1.000
5.5	♂	2	2	1	1	1	4	2	–	
	♀	2	2	1	1	1	4	2	–	1.000
6.5	♂	3	2	1	2	1	5	2	–	
	♀	3	2	1	2	1	6	2	–	0.878
7.5	♂	5	3	1	2	1	6	3	–	
	♀	6	3	1	2	1	6	3	–	0.959
8.5	♂	6	5	1	2	1	6	3	–	
	♀	6	5	2	2	1	6	3	–	0.878
9.5	♂	6	6	2	2	2	6	3	1	
	♀	6	6	2	2	2	6	3	1	1.000
10.5	♂	6	6	4	4	3	6	3	2	
	♀	6	6	5	4	3	6	4	1	0.798
11.5	♂	6	6	5	5	5	6	4	2	
	♀	6	6	5	6	5	6	4	1	0.721
12.5	♂	6	6	6	6	5	6	5	2	
	♀	6	6	6	6	6	6	4	2	0.959
13.5	♂	6	6	6	6	6	6	5	2	
	♀	6	6	6	6	6	6	6	2	0.721
14.5	♂	6	6	6	6	6	6	6	2	
	♀	6	6	6	6	6	6	6	2	1.000
15.5	♂	6	6	6	6	6	6	6	3	
	♀	6	6	6	6	6	6	6	3	1.000
16.5	♂	6	6	6	6	6	6	6	3	
	♀	6	6	6	6	6	6	6	3	1.000
17.5	♂	6	6	6	6	6	6	6	3	
	♀	6	6	6	6	6	6	6	4	0.959
18.5	♂	6	6	6	6	6	6	6	5	
	♀	6	6	6	6	6	6	6	4	0.759
19.5	♂	6	6	6	6	6	6	6	5	
	♀	6	6	6	6	6	6	6	5	1.000
20.5	♂	6	6	6	6	6	6	6	6	
	♀	6	6	6	6	6	6	6	6	1.000
21.5	♂	6	6	6	6	6	6	6	6	
	♀	6	6	6	6	6	6	6	6	1.000

Mandible										
Age*	Gender	LL1	LL2	LL3	LL4	LL5	LL6	LL7	LL8	*p*-value^^^
2.5	♂	2	2	1	1	–	3	–	–	
	♀	2	2	1	1	–	3	–	–	1.000
3.5	♂	2	1	1	1	1	3	1	–	
	♀	2	2	1	1	1	3	1	–	0.878
4.5	♂	2	2	1	1	1	3	1	–	
	♀	2	2	1	1	1	3	1	–	1.000
5.5	♂	2	2	1	1	1	4	1	–	
	♀	2	2	1	1	1	4	2	–	0.878
6.5	♂	5	3	1	1	1	5	2	–	
	♀	5	3	1	1	1	5	2	–	1.000
7.5	♂	5	5	1	2	1	6	2	–	
	♀	6	5	2	2	1	6	2	–	0.878
8.5	♂	6	5	2	2	1	6	3	–	
	♀	6	6	2	2	1	6	3	–	0.878
9.5	♂	6	6	2	2	1	6	3	1	
	♀	6	6	2	3	2	6	4	–	0.651
10.5	♂	6	6	5	4	2	6	4	1	
	♀	6	6	5	5	2	6	4	1	0.878
11.5	♂	6	6	6	5	3	6	5	2	
	♀	6	6	6	6	4	6	5	1	0.721
12.5	♂	6	6	6	6	6	6	5	2	
	♀	6	6	6	6	6	6	6	2	0.878
13.5	♂	6	6	6	6	6	6	5	3	
	♀	6	6	6	6	6	6	6	3	1.000
14.5	♂	6	6	6	6	6	6	5	3	
	♀	6	6	6	6	6	6	6	3	0.721
15.5	♂	6	6	6	6	6	6	5	3	
	♀	6	6	6	6	6	6	5	3	1.000
16.5	♂	6	6	6	6	6	6	6	4	
	♀	6	6	6	6	6	6	6	3	0.959
17.5	♂	6	6	6	6	6	6	6	4	
	♀	6	6	6	6	6	6	6	4	1.000
18.5	♂	6	6	6	6	6	6	6	5	
	♀	6	6	6	6	6	6	6	5	1.000
19.5	♂	6	6	6	6	6	6	6	6	
	♀	6	6	6	6	6	6	6	5	0.721
20.5	♂	6	6	6	6	6	6	6	6	
	♀	6	6	6	6	6	6	6	6	1.000
21.5	♂	6	6	6	6	6	6	6	6	
	♀	6	6	6	6	6	6	6	6	1.000

* *Age in midpoint of 1 year*.

♂ – *male*, ♀ - *female*.

^ *- Mann-Whitney U-test*.

*p - Level of significance*.

*–Data not available as the tooth did not exhibit signs of formation*.

For the children aged 2 to 14 years, the resorption pattern of primary teeth was more pronounced in the maxillary dentition compared to the mandibular dentition. At 2 years, all the primary teeth showed complete root development in males and except maxillary and mandibular canines that had an open apex. Both canines underwent root closure in the subsequent year. Difference in resorption between males and females was observed in 2, 4, 5, 8, 9, and 10 year-old children in the maxillary dentition and 2, 5, 6, and 10-year-old children in the mandibular dentition. However, Mann-Whitney *U*-test showed no statistically significant difference in the resorption pattern of primary teeth in males and females in all age ranges (*p* = 0.690 to *p* = 1.000), see Table 4.

**Table 4:** Resorption of maxillary and mandibular primary teeth of 2 to 13 years old Chinese females and males.

Maxilla											
Age*	Males					Females					
	i1	i2	c	m1	m2	i1	i2	c	m1	m2	*p*-value^
2.5	Ac	Ac	Ac	Ac	Ac	Ac	Ac	G	Ac	Ac	0.690
3.5	Ac	Ac	Ac	Ac	Ac	Ac	Ac	Ac	Ac	Ac	1.000
4.5	Ac	Ac	Ac	Ac	Ac	Res1/4	Ac	Ac	Ac	Ac	0.690
5.5	Ac	Ac	Ac	Ac	Ac	Res1/4	Ac	Ac	Ac	Ac	0.690
6.5	Res1/2	Res1/2	Ac	Ac	Ac	Res1/2	Res1/2	Ac	Ac	Ac	1.000
7.5	x	Res3/4	Ac	Ac	Ac	x	Res3/4	Ac	Ac	Ac	1.000
8.5	x	x	Res1/4	Ac	Ac	x	x	Res1/4	Res1/4	Ac	0.690
9.5	x	x	Res1/4	Res1/2	Res1/4	x	x	Res1/2	Res1/2	Res1/4	0.841
10.5	x	x	Res3/4	x	Res3/4	x	x	x	Res3/4	Res1/2	0.841
11.5	x	x	x	x	Res3/4	x	x	x	x	Res3/4	1.000
12.5	x	x	x	x	x	x	x	x	x	x	1.000
13.5	x	x	x	x	x	x	x	x	x	x	1.000
Mandible											
Age*	Males					Females					
	i1	i2	c	m1	m2	i1	i2	c	m1	m2	*p*-value^
2.5	Ac	Ac	Ac	Ac	Ac	Ac	Ac	G	Ac	Ac	0.690
3.5	Ac	Ac	Ac	Ac	Ac	Ac	Ac	Ac	Ac	Ac	1.000
4.5	Ac	Ac	Ac	Ac	Ac	Ac	Ac	Ac	Ac	Ac	1.000
5.5	Res3/4	Res1/2	Ac	Ac	Ac	Res1/2	Res1/2	Ac	Ac	Ac	0.841
6.5	x	Res3/4	Ac	Ac	Ac	x	Res1/2	Ac	Ac	Ac	0.841
7.5	x	x	Ac	Ac	Ac	x	x	Ac	Ac	Ac	1.000
8.5	x	x	Ac	Ac	Ac	x	x	Ac	Res1/4	Res1/4	0.690
9.5	x	x	Res1/2	Res1/2	Res1/4	x	x	Res1/2	Res1/2	Res1/4	1.000
10.5	x	x	x	x	Res1/2	x	x	Res3/4	X	Res3/4	0.690
11.5	x	x	x	x	Res3/4	x	x	x	x	Res3/4	1.000
12.5	x	x	x	x	x	x	x	x	x	x	1.000
13.5	x	x	x	x	x	x	x	x	x	x	1.000

* Age in midpoint of 1 year.

^ - Mann-Whitney *U*-test.

*p* - Level of significance.

G - Open Root apex.

x - No data available as the tooth underwent normal physiological exfoliation.

### 3.2 Construction of dental charts

**Figure 4:**
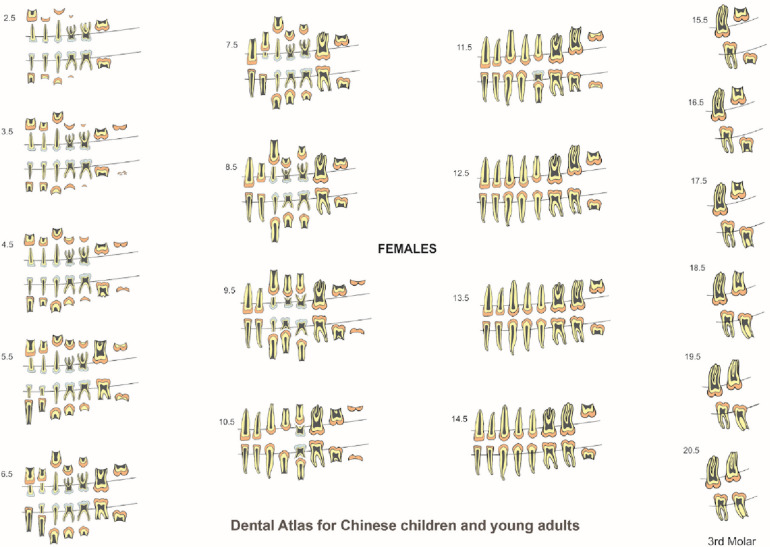
Dental atlas for Chinese females based on the formation and eruption of permanent teeth and resorption of primary teeth.

**Figure 5:**
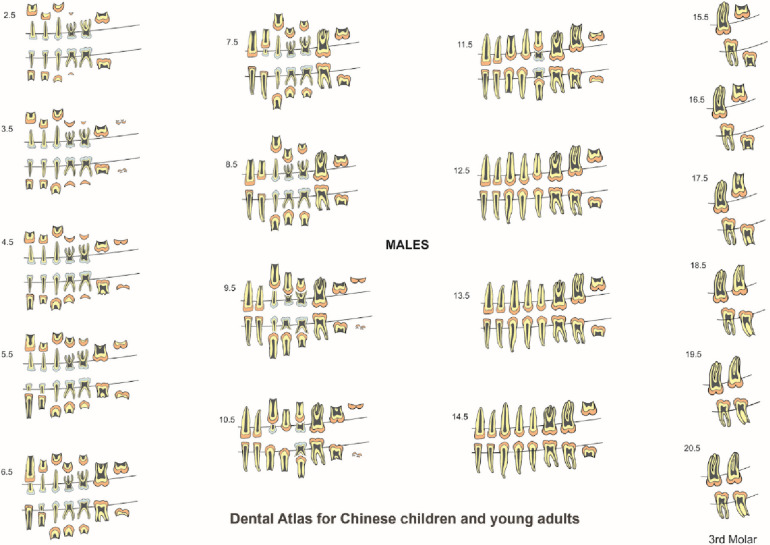
Dental atlas for Chinese males based on the formation and eruption of permanent teeth and resorption of primary teeth.

The formation and eruption patterns of permanent teeth and resorption pattern of primary teeth were different between males and females by one or two stages difference in most age ranges. Since dental development was different between the males and females, the data were presented as gender specific charts for females (Figure 4) and males (Figure 5). Templates of ten primary and sixteen permanent tooth morphology type (TMT) on the right side were obtained from a dental anatomy textbook (17). The images of teeth were carefully hand drawn and digitized using Adobe Photoshop software (Version CS6, Adobe Systems Ltd, Ireland). Internal tooth structures including enamel, dentin and pulp were colored appropriately to differentiate primary and permanent teeth. To illustrate different stages of dental formation, single tooth diagrams were prepared to exhibit eight stages of development resulting in a total of 128 images (16 TMT x 8 stages). For tooth resorption stages for primary tooth, three root resorption stages were incorporated yielding 30 images (10 TMT×3 stages). To construct dental charts, for each age range, images relating to the data for formation, and resorption were obtained. Individual images were then compiled to produce dental atlas charts for males and females aged 2 to 14 years. Since all permanent teeth except third molars completed development by 14 years, for ages 15 to 22 years, charts were constructed only for third molars. The images and charts were constructed by the first author (JJ) using Corel Draw Graphics software (CorelDRAW Graphics Suite X8, ON, Canada).

## 4 Discussion

Differences in dental emergence have been reported in different population groups. For example, advanced dental formation and emergence was reported in the WITS Atlas (12) based on black southern Africans compared to London Atlas that was based on Bangladeshi and Caucasian children (4). Dental age estimation was conducted using the London Atlas on mixed ethnic population in London and found to be accurate compared to Schour & Massler and Ubelaker charts (18). A similar finding was observed when the London Atlas was tested in Hispanic (19) as well as Iranian populations (20). In contrast, a study that tested the applicability of three dental charts including the London Atlas in New Zealand population found that all the charts demonstrated low accuracy and precision. This study further emphasized the need for population specific dental charts for accurate age estimation (21). Like population variations, sex difference in dental maturation was also an established phenomenon. Advanced dental emergence has been reported in girls compared to boys (22–24). However, a study that tested the accuracy of London Atlas in Portuguese population found difference between sexes and indicated the need for separate dental charts for each sex (25). In the present study, some difference was observed between the sexes; in females, both maxillary and mandibular first molars attained median stage of root closure at 8.5 years, one year earlier than in males. Similarly, canines and maxillary first pre-molars completed root development much earlier in females. In contrast, males showed advancement in the formation of maxillary third molar as the root apex closed at 19.5 years compared to 21.5 years in females. This trend was not observed in the mandibular third molar development, where root closure occurred around 21.5 years in both sex. Since the difference was not statistically significant, the data for males and females were combined and reported as a single dental atlas.

Eruption of permanent teeth is influenced by many other factors; socio-economic status, with their implicit connection with nutritional and health status (26), fluoride ingestion (27), and climate (28) have all been analyzed to possibly affect eruption timings. In contrast, a study conducted in southern Chinese children reported no significant difference in the eruption of teeth among children of low, medium and high socio-economic status (14). A similar finding was also observed in African Black children (29). Esan & Schepartz performed sample size calculation and found that a minimum sample size of 40 per age cohort was required to develop population specific dental atlas (12). In the present study, the data on dental maturation of permanent teeth from 2,306 subjects was re-used from the previous dental age study, accounting to over 80 subjects in each age cohort between 2 and 24 years of age (15). However, to analyze eruption of permanent teeth and resorption of primary teeth, we utilized only 40 samples per age cohort from the total sample. This sample size was relatively higher or equivalent to previous studies on dental atlas (4, 12).

The Chinese children in this study demonstrated advanced dental eruption in so much that all permanent teeth had erupted at 11.5 years whilst the London Atlas children had retained primary maxillary canines and both maxillary and mandibular second molars at this age. When the sex data were combined to compare the timing of eruption of the permanent teeth between the London Atlas and Chinese subjects in the current study, it was found that except for maxillary canines, all maxillary and mandibular incisors erupted earlier in subjects in the London Atlas. In contrast, pre-molars erupted earlier in Chinese children; however, first and second molars were similar between these groups. Both London Atlas and Chinese subjects had half of the roots of third molars formed at 16.5 years, whilst the Black African children demonstrated similar stage at 14.5 years. The root closure of third molars occurred at 17.5 years in Black African subjects whilst it happened at 21.5 years in Chinese and London Atlas subjects. These findings suggest the possibility of population variations in dental formation in third molars between the London Atlas, African Black and the Chinese subjects (4, 12). Comparison of dental development between the London Atlas and the WITS Atlas for each age cohort had been already reported (12). Another area of discussion is influence of secular trends in dental development. It has been shown that children born in different centuries (30) and between few decades (31, 32) have different patterns of dental development. This trend has also been observed in 5–6 years old Chinese children (33). To address this issue, in the current study, we utilized only radiographs belonging to modern samples. This study was conducted in 2012 and the search criteria was restricted to include only the most recent samples to represent modern day children, adolescents and young adults. The age of subjects ranged from 2 to 24 years and the date of birth of the subjects were between 1985 and 2010. For example, for 5-year old children, those who were born around 2005 alone were obtained and a similar search strategy was extended to all other age ranges.

Standards for emergence of permanent teeth in southern Chinese children were first reported in 1965 (12). This study was based on clinical observation and hence did not include other aspects of dental development including the resorption of primary teeth and formation pattern of permanent teeth at subsequent years. In our study, all the parameters of tooth formation and eruption were assessed from radiographs. It has been reported that early loss of primary molars delays the eruption of the premolars and conversely, delayed exfoliation results in earlier eruption of the premolars (34). Accelerated eruption of premolars beneath the pulpotomized primary molars have also been reported (35). In addition, trauma (36), odontogenic cysts (37), odontogenic tumors (38), gingival enlargements (39), and supernumerary tooth (40) has been reported to influence dental formation and emergence. Consequently, subjects with severe dental anomalies and other forms of development disorders were excluded from the analysis. It is evident that pathologically affected primary teeth influences the eruption of permanent teeth; however, the relationship between the pathologically involved primary teeth and the stage of formation of permanent teeth has not been established yet (34).

The method in which median stages of dental development analyzed in this study was like a study conducted in the London Atlas study (4). For subjects aged 2 to 14 years, the entire complement of maxillary and mandibular dentition was displayed in the atlas. Since all the teeth had attained complete development by 14 years, for age range of 15 to 20 years, we have presented only maxillary and mandibular third molars. This is like the London Atlas that had full dentition until 15 years followed by third molars in the 16 to 23 years range. Radiographs of subjects used in this study represent a sample population of Chinese ethnic group living in Hong Kong. They are identified by the uniqueness of their name and the family details recorded in the patient files. All the radiographs were primarily taken to assist in clinical diagnosis and were hence re-used in the current study. It is to be noted that panoramic radiographs for young patients below 5 years of age were obtained only following strong indication to assist in diagnosis and treatment planning. Occasionally, they were also taken on uncooperative children who resist to taking conventional intraoral radiographs.

Demirjian’s 8 stage method was used in our study considering the reliability and ease in identification of stages. This staging method was like the WITS Atlas study (12), however, The London Atlas study utilized 13 stage method (4). To evaluate initial stages of development of primary teeth, investigators must rely on skeletonized or preserved human remains with known details of birth and death. The London Atlas study comprised of skeletal samples as young as 30 weeks in-utero that were obtained from Maurice Stack collection at the Royal College of Surgeons of England (4). In current study, archived samples of modern Chinese subjects aged 4 months to 2 years old could not be found and so, it was impossible to present data corresponding to this age. Moreover, it has been shown that the London dental atlas tends to overestimate the age (41). Following this, a recent review questioned the logic of using dental atlas for age estimation. It reviewed five dental atlases and found inconsistency I the study design, statistical procedures and presentation styles (42). This dental atlas should be used with caution for the purpose of dental age estimation as the age is segregated by the mid-point of one year. Moreover, this atlas has not been subjected to blind validation analysis. For accurate estimation of age, it is recommended to use simple average method (SAM) as described in the author’s previous article (15).

## 5 Conclusions

We have presented evidence based dental chart developed from a large sample of children and young adults of Chinese ethnicity. This dental atlas serves as a practical tool for age estimation in forensic investigations, assist in clinical diagnosis and treatment planning as well as indicators of developments in public health. Future research should focus on generating population specific data on dental development as well as integrating newer imaging technologies.

## Data Availability

The original contributions presented in the study are included in the article/Supplementary Material, further inquiries can be directed to the corresponding author.
