# Corrigendum: Development and eruption of human teeth in the Chinese population: a comprehensive dental atlas

**DOI:** 10.3389/fdmed.2024.1504277

**Published:** 2024-11-25

**Authors:** Jayakumar Jayaraman

**Affiliations:** Department of Pediatric Dentistry, Virginia Commonwealth University School of Dentistry, Richmond, VA, United States

**Keywords:** dental atlas, Chinese, dental development, dental chart, primary teeth, permanent teeth, human dentition, forensics age estimation

A Corrigendum on Development and eruption of human teeth in the Chinese population: a comprehensive dental atlas By Jayaraman J. (2024). Front. Dent. Med. 5:1434417. doi: 10.3389/fdmed.2024.1434417

In the published article, there was an error in Figures 4 and 5 as published. The images for these figures did not correspond to the captions: Figure 4 should correspond to dental atlas for Females and Figure 5 should correspond to dental atlas for Males. The correct Figures 4 and 5 and their captions appear below.

**Figure 4:**
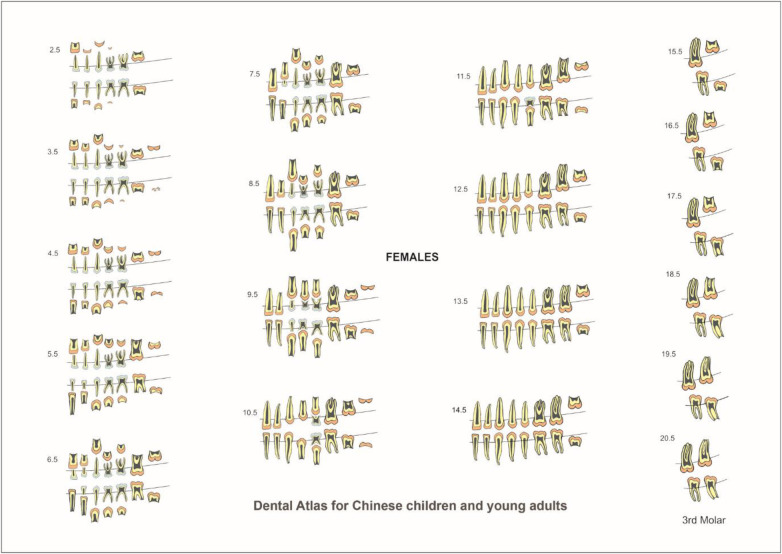
Dental atlas for Chinese females based on the formation and eruption of permanent teeth and resorption of primary teeth.

**Figure 5:**
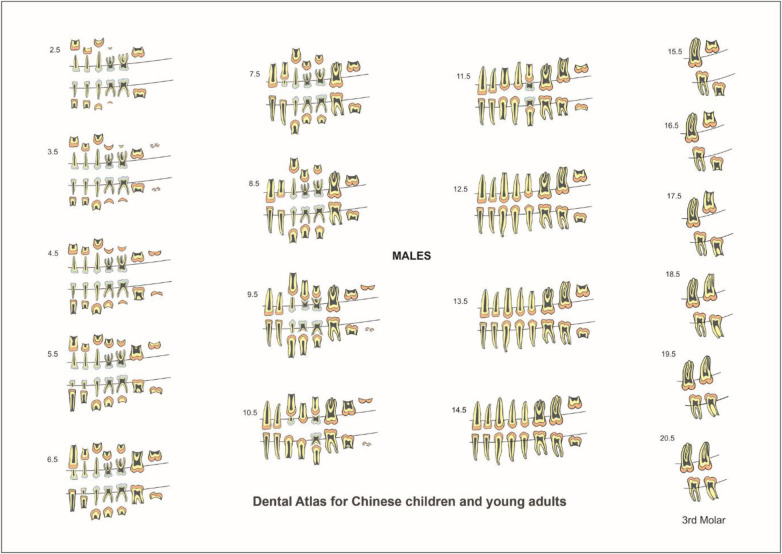
Dental atlas for Chinese males based on the formation and eruption of permanent teeth and resorption of primary teeth.

The authors apologize for this error and state that this does not change the scientific conclusions of the article in any way. The original article has been updated.

