# Supplementary material for: Development and eruption of human teeth in the Chinese population: a comprehensive dental atlas
Source: Front Dent Med. 2024 Aug 27;5:1434417. doi: 10.3389/fdmed.2024.1434417 (PMC11797833; doi:10.3389/fdmed.2024.1434417)
Supplement: Supplementary file 1 [file Table1.docx]

**Table S1.** Summary data of tooth formation stages for Han Chinese children and young adults.

**Table S1a.**  Summary data of maxillary left central incisor (ULI f & m)

| TDS | n | mean | sd | se | med | min | max | range | 99%CIL | 99%CIU | 0.5%ile | 5%ile | 10%ile | 25%ile | 50%ile | 75%ile | 90%ile | 95%ile | 99.5%ile | TDS |
| --- | --- | --- | --- | --- | --- | --- | --- | --- | --- | --- | --- | --- | --- | --- | --- | --- | --- | --- | --- | --- |
| **UL1Af** | **-** | **-** | **-** | **-** | **-** | **-** | **-** | **-** | **-** | **-** | **-** | **-** | **-** | **-** | **-** | **-** | **-** | **-** | **-** | **UL1Af** |
| **UL1Am** | **-** | **-** | **-** | **-** | **-** | **-** | **-** | **-** | **-** | **-** | **-** | **-** | **-** | **-** | **-** | **-** | **-** | **-** | **-** | **UL1Am** |
| **UL1Bf** | **-** | **-** | **-** | **-** | **-** | **-** | **-** | **-** | **-** | **-** | **-** | **-** | **-** | **-** | **-** | **-** | **-** | **-** | **-** | **UL1Bf** |
| **UL1Bm** | **-** | **-** | **-** | **-** | **-** | **-** | **-** | **-** | **-** | **-** | **-** | **-** | **-** | **-** | **-** | **-** | **-** | **-** | **-** | **UL1Bm** |
| **UL1Cf** | **-** | **-** | **-** | **-** | **-** | **-** | **-** | **-** | **-** | **-** | **-** | **-** | **-** | **-** | **-** | **-** | **-** | **-** | **-** | **UL1Cf** |
| **UL1Cm** | **-** | **-** | **-** | **-** | **-** | **-** | **-** | **-** | **-** | **-** | **-** | **-** | **-** | **-** | **-** | **-** | **-** | **-** | **-** | **UL1Cm** |
| **UL1Df** | 148 | 3.81 | 1.03 | 0.08 | 3.78 | 2.13 | 6.31 | 4.18 | 3.59 | 4.03 | 2.16 | 2.33 | 2.44 | 2.94 | 3.78 | 4.57 | 5.19 | 5.56 | 6.21 | **UL1Df** |
| **UL1Dm** | 169 | 4.03 | 1.13 | 0.09 | 3.92 | 2.17 | 7.15 | 4.98 | 3.81 | 4.26 | 2.31 | 2.50 | 2.58 | 2.97 | 3.92 | 4.85 | 5.60 | 5.99 | 6.99 | **UL1Dm** |
| **UL1Ef** | 120 | 5.56 | 0.74 | 0.07 | 5.56 | 3.28 | 7.69 | 4.41 | 5.38 | 5.73 | 3.58 | 3.97 | 4.90 | 5.12 | 5.56 | 5.98 | 6.38 | 6.53 | 7.68 | **UL1Ef** |
| **UL1Em** | 96 | 5.74 | 0.70 | 0.07 | 5.62 | 3.82 | 8.03 | 4.21 | 5.56 | 5.92 | 4.21 | 4.86 | 5.06 | 5.23 | 5.62 | 6.05 | 6.55 | 6.97 | 7.96 | **UL1Em** |
| **UL1Ff** | 112 | 7.50 | 1.01 | 0.10 | 7.55 | 5.18 | 10.01 | 4.83 | 7.25 | 7.74 | 5.24 | 5.69 | 6.08 | 6.86 | 7.55 | 8.10 | 8.63 | 9.25 | 9.99 | **UL1Ff** |
| **UL1Fm** | 122 | 7.68 | 1.13 | 0.10 | 7.68 | 4.42 | 10.28 | 5.86 | 7.42 | 7.95 | 5.14 | 6.02 | 6.24 | 6.86 | 7.68 | 8.42 | 9.23 | 9.45 | 10.19 | **UL1Fm** |
| **UL1Gf** | 59 | 9.42 | 1.09 | 0.14 | 9.35 | 7.21 | 12.18 | 4.97 | 9.05 | 9.79 | 7.25 | 7.98 | 8.24 | 8.58 | 9.35 | 9.95 | 10.96 | 11.21 | 12.16 | **UL1Gf** |
| **UL1Gm** | 69 | 9.92 | 1.25 | 0.15 | 9.78 | 7.70 | 12.73 | 5.03 | 9.53 | 10.30 | 7.71 | 8.09 | 8.20 | 8.92 | 9.78 | 10.60 | 11.64 | 12.19 | 12.73 | **UL1Gm** |
| **UL1Hf** | 64 | 10.00 | 0.86 | 0.11 | 10.25 | 7.86 | 10.94 | 3.08 | 9.72 | 10.28 | 7.90 | 8.41 | 8.79 | 9.33 | 10.25 | 10.73 | 10.87 | 10.91 | 10.94 | **UL1Hf** |
| **UL1Hm** | 116 | 11.34 | 0.99 | 0.09 | 11.41 | 8.50 | 12.78 | 4.28 | 11.10 | 11.57 | 8.77 | 9.46 | 10.13 | 10.77 | 11.41 | 12.06 | 12.56 | 12.70 | 12.77 | **UL1Hm** |
| TDS | n | mean | sd | se | med | min | max | range | 99%CIL | 99%CIU | 0.5%ile | 5%ile | 10%ile | 25%ile | 50%ile | 75%ile | 90%ile | 95%ile | 99.5%ile | TDS |

**Table S1b.** Summary data of maxillary left lateral incisor (UL2 f & m)

| TDS | n | mean | sd | se | med | min | max | range | 99%CIL | 99%CIU | 0.5%ile | 5%ile | 10%ile | 25%ile | 50%ile | 75%ile | 90%ile | 95%ile | 99.5%ile | TDS |
| --- | --- | --- | --- | --- | --- | --- | --- | --- | --- | --- | --- | --- | --- | --- | --- | --- | --- | --- | --- | --- |
| **UL2Af** | **-** | **-** | **-** | **-** | **-** | **-** | **-** | **-** | **-** | **-** | **-** | **-** | **-** | **-** | **-** | **-** | **-** | **-** | **-** | **UL2Af** |
| **UL2Am** | **-** | **-** | **-** | **-** | **-** | **-** | **-** | **-** | **-** | **-** | **-** | **-** | **-** | **-** | **-** | **-** | **-** | **-** | **-** | **UL2Am** |
| **UL2Bf** | **-** | **-** | **-** | **-** | **-** | **-** | **-** | **-** | **-** | **-** | **-** | **-** | **-** | **-** | **-** | **-** | **-** | **-** | **-** | **UL2Bf** |
| **UL2Bm** | **-** | **-** | **-** | **-** | **-** | **-** | **-** | **-** | **-** | **-** | **-** | **-** | **-** | **-** | **-** | **-** | **-** | **-** | **-** | **UL2Bm** |
| **UL2Cf** | 40 | 2.97 | 0.64 | 0.10 | 2.83 | 2.17 | 4.25 | 2.08 | 2.71 | 3.23 | 2.18 | 2.28 | 2.32 | 2.42 | 2.83 | 3.26 | 4.08 | 4.23 | 4.25 | **UL2Cf** |
| **UL2Cm** | 44 | 2.97 | 0.51 | 0.08 | 2.84 | 2.17 | 4.42 | 2.25 | 2.78 | 3.17 | 2.22 | 2.43 | 2.50 | 2.55 | 2.84 | 3.31 | 3.71 | 3.83 | 4.32 | **UL2Cm** |
| **UL2Df** | 148 | 4.50 | 1.01 | 0.08 | 4.55 | 2.66 | 7.69 | 5.03 | 4.29 | 4.71 | 2.70 | 2.93 | 3.22 | 3.76 | 4.55 | 5.17 | 5.88 | 6.17 | 6.73 | **UL2Df** |
| **UL2Dm** | 177 | 4.87 | 1.04 | 0.08 | 5.00 | 2.34 | 7.15 | 4.81 | 4.67 | 5.07 | 2.43 | 2.94 | 3.54 | 4.11 | 5.00 | 5.60 | 6.18 | 6.42 | 6.98 | **UL2Dm** |
| **UL2Ef** | 109 | 6.12 | 0.88 | 0.08 | 5.81 | 4.67 | 8.72 | 4.05 | 5.90 | 6.34 | 4.84 | 5.09 | 5.18 | 5.52 | 5.81 | 6.61 | 7.57 | 7.92 | 8.64 | **UL2Ef** |
| **UL2Em** | 95 | 6.59 | 0.98 | 0.10 | 6.49 | 4.42 | 8.92 | 4.50 | 6.33 | 6.85 | 4.71 | 5.21 | 5.41 | 5.92 | 6.49 | 7.28 | 8.07 | 8.25 | 8.88 | **UL2Em** |
| **UL2Ff** | 96 | 7.91 | 0.90 | 0.09 | 7.85 | 5.69 | 10.01 | 4.31 | 7.67 | 8.14 | 5.79 | 6.47 | 6.80 | 7.38 | 7.85 | 8.47 | 9.18 | 9.37 | 9.85 | **UL2Ff** |
| **UL2Fm** | 90 | 8.59 | 1.16 | 0.12 | 8.37 | 5.60 | 12.73 | 7.13 | 8.27 | 8.90 | 5.99 | 7.18 | 7.39 | 7.84 | 8.37 | 9.23 | 9.93 | 10.46 | 12.40 | **UL2Fm** |
| **UL2Gf** | 63 | 10.12 | 1.42 | 0.18 | 9.82 | 7.86 | 14.39 | 6.52 | 9.66 | 10.58 | 7.98 | 8.47 | 8.54 | 9.27 | 9.82 | 10.56 | 12.22 | 12.98 | 14.15 | **UL2Gf** |
| **UL2Gm** | 58 | 10.52 | 1.22 | 0.16 | 10.31 | 8.50 | 13.97 | 5.48 | 10.11 | 10.94 | 8.51 | 8.90 | 9.13 | 9.65 | 10.31 | 11.42 | 12.20 | 12.74 | 13.67 | **UL2Gm** |
| **UL2Hf** | 46 | 10.41 | 0.59 | 0.09 | 10.59 | 8.79 | 11.13 | 2.34 | 10.18 | 10.63 | 8.82 | 9.04 | 9.64 | 10.20 | 10.59 | 10.85 | 10.92 | 10.94 | 11.10 | **UL2Hf** |
| **UL2Hm** | 69 | 11.07 | 0.66 | 0.08 | 11.15 | 8.97 | 11.98 | 3.01 | 10.86 | 11.27 | 9.02 | 10.13 | 10.21 | 10.64 | 11.15 | 11.57 | 11.87 | 11.91 | 11.98 | **UL2Hm** |
| TDS | n | mean | sd | se | med | min | max | range | 99%CIL | 99%CIU | 0.5%ile | 5%ile | 10%ile | 25%ile | 50%ile | 75%ile | 90%ile | 95%ile | 99.5%ile | TDS |

**Table S1c.** Summary data of maxillary left canine (UL3 f & m)

| TDS | n | mean | sd | se | med | min | max | range | 99%CIL | 99%CIU | 0.5%ile | 5%ile | 10%ile | 25%ile | 50%ile | 75%ile | 90%ile | 95%ile | 99.5%ile | TDS |
| --- | --- | --- | --- | --- | --- | --- | --- | --- | --- | --- | --- | --- | --- | --- | --- | --- | --- | --- | --- | --- |
| **UL3Af** | **-** | **-** | **-** | **-** | **-** | **-** | **-** | **-** | **-** | **-** | **-** | **-** | **-** | **-** | **-** | **-** | **-** | **-** | **-** | **UL3Af** |
| **UL3Am** | **-** | **-** | **-** | **-** | **-** | **-** | **-** | **-** | **-** | **-** | **-** | **-** | **-** | **-** | **-** | **-** | **-** | **-** | **-** | **UL3Am** |
| **UL3Bf** | **-** | **-** | **-** | **-** | **-** | **-** | **-** | **-** | **-** | **-** | **-** | **-** | **-** | **-** | **-** | **-** | **-** | **-** | **-** | **UL3Bf** |
| **UL3Bm** | **-** | **-** | **-** | **-** | **-** | **-** | **-** | **-** | **-** | **-** | **-** | **-** | **-** | **-** | **-** | **-** | **-** | **-** | **-** | **UL3Bm** |
| **UL3Cf** | 61 | 2.99 | 0.62 | 0.08 | 2.90 | 2.13 | 5.07 | 2.94 | 2.78 | 3.19 | 2.13 | 2.20 | 2.33 | 2.46 | 2.90 | 3.29 | 3.89 | 4.20 | 4.84 | **UL3Cf** |
| **UL3Cm** | 68 | 3.26 | 0.85 | 0.10 | 2.97 | 2.17 | 6.15 | 3.98 | 2.99 | 3.53 | 2.23 | 2.43 | 2.50 | 2.65 | 2.97 | 3.64 | 4.58 | 5.15 | 5.93 | **UL3Cm** |
| **UL3Df** | 181 | 4.93 | 1.01 | 0.08 | 5.01 | 2.45 | 8.64 | 6.18 | 4.73 | 5.12 | 2.70 | 3.24 | 3.58 | 4.23 | 5.01 | 5.59 | 6.14 | 6.31 | 8.11 | **UL3Df** |
| **UL3Dm** | 208 | 5.36 | 0.98 | 0.07 | 5.40 | 3.24 | 8.15 | 4.90 | 5.19 | 5.54 | 3.25 | 3.80 | 3.91 | 4.71 | 5.40 | 5.98 | 6.57 | 7.08 | 7.57 | **UL3Dm** |
| **UL3Ef** | 100 | 6.83 | 1.09 | 0.11 | 6.79 | 5.10 | 10.37 | 5.27 | 6.55 | 7.11 | 5.12 | 5.40 | 5.56 | 5.81 | 6.79 | 7.68 | 8.17 | 8.47 | 10.03 | **UL3Ef** |
| **UL3Em** | 97 | 7.77 | 1.00 | 0.10 | 7.85 | 5.66 | 10.26 | 4.60 | 7.50 | 8.03 | 5.79 | 6.01 | 6.39 | 7.23 | 7.85 | 8.39 | 8.91 | 9.31 | 10.08 | **UL3Em** |
| **UL3Ff** | 129 | 9.09 | 1.32 | 0.12 | 9.11 | 6.59 | 13.03 | 6.44 | 8.79 | 9.39 | 6.69 | 7.27 | 7.50 | 8.10 | 9.11 | 9.94 | 10.80 | 11.54 | 12.64 | **UL3Ff** |
| **UL3Fm** | 141 | 10.32 | 1.30 | 0.11 | 10.22 | 6.99 | 13.13 | 6.14 | 10.04 | 10.60 | 7.41 | 8.13 | 8.70 | 9.39 | 10.22 | 11.37 | 11.99 | 12.52 | 13.10 | **UL3Fm** |
| **UL3Gf** | 105 | 12.11 | 1.74 | 0.17 | 11.99 | 9.01 | 17.64 | 8.63 | 11.67 | 12.55 | 9.07 | 9.78 | 10.04 | 10.83 | 11.99 | 13.05 | 14.42 | 14.85 | 17.28 | **UL3Gf** |
| **UL3Gm** | 83 | 12.68 | 1.31 | 0.14 | 12.73 | 10.12 | 15.76 | 5.65 | 12.31 | 13.05 | 10.27 | 10.76 | 11.07 | 11.53 | 12.73 | 13.47 | 14.48 | 14.97 | 15.76 | **UL3Gm** |
| **UL3Hf** | 108 | 13.31 | 0.98 | 0.09 | 13.44 | 11.13 | 14.82 | 3.69 | 13.07 | 13.56 | 11.21 | 11.59 | 11.85 | 12.59 | 13.44 | 14.09 | 14.60 | 14.70 | 14.82 | **UL3Hf** |
| **UL3Hm** | 100 | 14.23 | 0.88 | 0.09 | 14.24 | 11.58 | 15.60 | 4.03 | 14.00 | 14.46 | 11.67 | 12.68 | 13.08 | 13.77 | 14.24 | 14.90 | 15.38 | 15.50 | 15.60 | **UL3Hm** |
| TDS | n | mean | sd | se | med | min | max | range | 99%CIL | 99%CIU | 0.5%ile | 5%ile | 10%ile | 25%ile | 50%ile | 75%ile | 90%ile | 95%ile | 99.5%ile | TDS |

**Table S1d.** Summary data of maxillary left first pre-molar (UL4 f & m)

| TDS | n | mean | sd | se | med | min | max | range | 99%CIL | 99%CIU | 0.5%ile | 5%ile | 10%ile | 25%ile | 50%ile | 75%ile | 90%ile | 95%ile | 99.5%ile | TDS |
| --- | --- | --- | --- | --- | --- | --- | --- | --- | --- | --- | --- | --- | --- | --- | --- | --- | --- | --- | --- | --- |
| **UL4Af** | 13 | 2.98 | 0.24 | 0.07 | 2.92 | 2.66 | 3.43 | 0.77 | 2.81 | 3.15 | 2.66 | 2.70 | 2.74 | 2.79 | 2.92 | 3.21 | 3.26 | 3.33 | 3.42 | **UL4Af** |
| **UL4Am** | 11 | 3.09 | 0.49 | 0.15 | 2.93 | 2.54 | 3.92 | 1.38 | 2.71 | 3.47 | 2.54 | 2.54 | 2.55 | 2.81 | 2.93 | 3.31 | 3.85 | 3.88 | 3.91 | **UL4Am** |
| **UL4Bf** | 52 | 3.86 | 0.58 | 0.08 | 3.80 | 2.90 | 6.18 | 3.28 | 3.65 | 4.07 | 2.91 | 3.10 | 3.25 | 3.47 | 3.80 | 4.16 | 4.54 | 4.74 | 5.85 | **UL4Bf** |
| **UL4Bm** | 61 | 4.03 | 0.57 | 0.07 | 4.03 | 2.44 | 4.96 | 2.52 | 3.84 | 4.21 | 2.51 | 2.97 | 3.33 | 3.74 | 4.03 | 4.43 | 4.77 | 4.82 | 4.96 | **UL4Bm** |
| **UL4Cf** | 63 | 4.75 | 0.64 | 0.08 | 4.76 | 2.95 | 6.38 | 3.43 | 4.54 | 4.96 | 3.09 | 3.68 | 3.91 | 4.38 | 4.76 | 5.13 | 5.33 | 5.82 | 6.32 | **UL4Cf** |
| **UL4Cm** | 80 | 5.16 | 0.70 | 0.08 | 5.19 | 3.24 | 6.60 | 3.36 | 4.95 | 5.36 | 3.31 | 3.80 | 4.19 | 4.80 | 5.19 | 5.60 | 6.02 | 6.15 | 6.51 | **UL4Cm** |
| **UL4Df** | 190 | 6.52 | 1.15 | 0.08 | 6.19 | 4.30 | 10.37 | 6.08 | 6.31 | 6.74 | 4.87 | 5.09 | 5.26 | 5.60 | 6.19 | 7.50 | 8.18 | 8.43 | 9.71 | **UL4Df** |
| **UL4Dm** | 171 | 6.82 | 1.22 | 0.09 | 6.53 | 4.42 | 10.26 | 5.84 | 6.58 | 7.06 | 4.91 | 5.17 | 5.44 | 5.85 | 6.53 | 7.63 | 8.70 | 9.03 | 9.94 | **UL4Dm** |
| **UL4Ef** | 53 | 8.29 | 1.05 | 0.14 | 8.46 | 5.80 | 10.06 | 4.26 | 7.92 | 8.66 | 5.89 | 6.64 | 6.99 | 7.56 | 8.46 | 9.17 | 9.50 | 9.91 | 10.05 | **UL4Ef** |
| **UL4Em** | 52 | 8.62 | 0.87 | 0.12 | 8.62 | 6.62 | 10.62 | 4.00 | 8.31 | 8.93 | 6.65 | 7.31 | 7.70 | 8.04 | 8.62 | 9.21 | 9.61 | 10.05 | 10.54 | **UL4Em** |
| **UL4Ff** | 73 | 10.18 | 1.21 | 0.14 | 10.06 | 7.79 | 13.51 | 5.71 | 9.81 | 10.54 | 7.86 | 8.48 | 8.70 | 9.34 | 10.06 | 10.91 | 11.80 | 12.42 | 13.33 | **UL4Ff** |
| **UL4Fm** | 86 | 10.59 | 1.17 | 0.13 | 10.41 | 8.60 | 14.86 | 6.26 | 10.27 | 10.92 | 8.68 | 9.10 | 9.21 | 9.68 | 10.41 | 11.37 | 11.86 | 12.54 | 14.49 | **UL4Fm** |
| **UL4Gf** | 73 | 11.91 | 1.40 | 0.16 | 11.99 | 8.85 | 14.87 | 6.02 | 11.49 | 12.34 | 9.11 | 9.95 | 10.24 | 10.83 | 11.99 | 12.70 | 13.90 | 14.29 | 14.85 | **UL4Gf** |
| **UL4Gm** | 74 | 12.14 | 1.09 | 0.13 | 12.04 | 9.88 | 15.58 | 5.70 | 11.81 | 12.47 | 10.08 | 10.56 | 10.80 | 11.35 | 12.04 | 12.90 | 13.58 | 13.74 | 15.17 | **UL4Gm** |
| **UL4Hf** | 84 | 12.84 | 0.80 | 0.09 | 13.00 | 10.72 | 14.06 | 3.33 | 12.62 | 13.07 | 10.89 | 11.45 | 11.68 | 12.35 | 13.00 | 13.48 | 13.77 | 13.93 | 14.04 | **UL4Hf** |
| **UL4Hm** | 64 | 13.31 | 0.78 | 0.10 | 13.52 | 10.92 | 14.19 | 3.27 | 13.06 | 13.56 | 11.07 | 11.79 | 12.23 | 12.82 | 13.52 | 13.95 | 14.05 | 14.18 | 14.19 | **UL4Hm** |
| TDS | n | mean | sd | se | med | min | max | range | 99%CIL | 99%CIU | 0.5%ile | 5%ile | 10%ile | 25%ile | 50%ile | 75%ile | 90%ile | 95%ile | 99.5%ile | TDS |

**Table S1e.** Summary data of maxillary left second pre-molar (UL5 f & m)

| TDS | n | mean | sd | se | med | min | max | range | 99%CIL | 99%CIU | 0.5%ile | 5%ile | 10%ile | 25%ile | 50%ile | 75%ile | 90%ile | 95%ile | 99.5%ile | TDS |
| --- | --- | --- | --- | --- | --- | --- | --- | --- | --- | --- | --- | --- | --- | --- | --- | --- | --- | --- | --- | --- |
| **UL5Af** | 11 | 3.89 | 0.60 | 0.18 | 3.91 | 2.86 | 4.90 | 2.04 | 3.43 | 4.36 | 2.89 | 3.13 | 3.40 | 3.51 | 3.91 | 4.25 | 4.62 | 4.76 | 4.88 | **UL5Af** |
| **UL5Am** | 21 | 4.22 | 0.47 | 0.10 | 4.17 | 3.59 | 5.12 | 1.53 | 3.96 | 4.48 | 3.60 | 3.66 | 3.73 | 3.81 | 4.17 | 4.62 | 4.77 | 5.10 | 5.12 | **UL5Am** |
| **UL5Bf** | 57 | 4.61 | 0.56 | 0.07 | 4.67 | 2.90 | 5.68 | 2.78 | 4.42 | 4.80 | 3.06 | 3.76 | 3.90 | 4.25 | 4.67 | 4.99 | 5.24 | 5.49 | 5.65 | **UL5Bf** |
| **UL5Bm** | 47 | 4.66 | 0.68 | 0.10 | 4.71 | 3.24 | 6.15 | 2.90 | 4.40 | 4.91 | 3.28 | 3.63 | 3.82 | 4.24 | 4.71 | 4.98 | 5.62 | 5.87 | 6.12 | **UL5Bm** |
| **UL5Cf** | 75 | 5.62 | 0.70 | 0.08 | 5.59 | 2.95 | 8.05 | 5.10 | 5.41 | 5.83 | 3.43 | 4.74 | 5.05 | 5.17 | 5.59 | 6.08 | 6.36 | 6.68 | 7.68 | **UL5Cf** |
| **UL5Cm** | 79 | 5.64 | 0.66 | 0.07 | 5.52 | 3.47 | 7.15 | 3.68 | 5.45 | 5.83 | 3.84 | 4.70 | 5.07 | 5.17 | 5.52 | 6.09 | 6.55 | 6.76 | 7.08 | **UL5Cm** |
| **UL5Df** | 125 | 6.98 | 1.07 | 0.10 | 7.06 | 5.17 | 9.67 | 4.51 | 6.73 | 7.23 | 5.21 | 5.43 | 5.57 | 5.92 | 7.06 | 7.70 | 8.25 | 8.63 | 9.57 | **UL5Df** |
| **UL5Dm** | 134 | 7.15 | 1.21 | 0.10 | 7.23 | 5.00 | 10.06 | 5.06 | 6.88 | 7.42 | 5.07 | 5.38 | 5.66 | 6.10 | 7.23 | 8.08 | 8.83 | 9.23 | 9.95 | **UL5Dm** |
| **UL5Ef** | 49 | 8.55 | 0.87 | 0.12 | 8.57 | 6.14 | 10.06 | 3.92 | 8.23 | 8.87 | 6.22 | 7.36 | 7.47 | 8.09 | 8.57 | 9.19 | 9.69 | 9.93 | 10.05 | **UL5Ef** |
| **UL5Em** | 55 | 8.65 | 1.02 | 0.14 | 8.76 | 5.97 | 11.27 | 5.30 | 8.30 | 9.01 | 6.11 | 7.07 | 7.49 | 7.97 | 8.76 | 9.24 | 9.84 | 10.34 | 11.09 | **UL5Em** |
| **UL5Ff** | 99 | 10.56 | 1.34 | 0.14 | 10.30 | 7.63 | 14.72 | 7.09 | 10.21 | 10.90 | 7.71 | 8.66 | 9.10 | 9.68 | 10.30 | 11.45 | 12.23 | 12.84 | 14.38 | **UL5Ff** |
| **UL5Fm** | 104 | 10.86 | 1.17 | 0.11 | 10.75 | 8.60 | 14.86 | 6.26 | 10.56 | 11.15 | 8.70 | 9.21 | 9.56 | 9.97 | 10.75 | 11.68 | 12.09 | 13.06 | 14.41 | **UL5Fm** |
| **UL5Gf** | 82 | 12.57 | 1.49 | 0.16 | 12.53 | 8.85 | 16.36 | 7.51 | 12.14 | 12.99 | 9.15 | 10.37 | 10.73 | 11.52 | 12.53 | 13.39 | 14.44 | 14.86 | 16.33 | **UL5Gf** |
| **UL5Gm** | 88 | 12.78 | 1.28 | 0.14 | 12.71 | 10.23 | 16.41 | 6.18 | 12.43 | 13.13 | 10.33 | 10.99 | 11.26 | 11.89 | 12.71 | 13.54 | 14.45 | 15.23 | 16.12 | **UL5Gm** |
| **UL5Hf** | 58 | 13.11 | 0.70 | 0.09 | 13.23 | 11.50 | 14.10 | 2.60 | 12.88 | 13.35 | 11.50 | 11.80 | 12.13 | 12.62 | 13.23 | 13.64 | 13.95 | 14.06 | 14.10 | **UL5Hf** |
| **UL5Hm** | 51 | 13.49 | 0.75 | 0.10 | 13.71 | 11.39 | 14.25 | 2.86 | 13.22 | 13.76 | 11.42 | 11.79 | 12.32 | 13.14 | 13.71 | 14.02 | 14.19 | 14.23 | 14.25 | **UL5Hm** |
| TDS | n | mean | sd | se | med | min | max | range | 99%CIL | 99%CIU | 0.5%ile | 5%ile | 10%ile | 25%ile | 50%ile | 75%ile | 90%ile | 95%ile | 99.5%ile | TDS |

**Table S1f.** Summary data of maxillary left first molar (UL6 f & m)

| TDS | n | mean | sd | se | med | min | max | range | 99%CIL | 99%CIU | 0.5%ile | 5%ile | 10%ile | 25%ile | 50%ile | 75%ile | 90%ile | 95%ile | 99.5%ile | TDS |
| --- | --- | --- | --- | --- | --- | --- | --- | --- | --- | --- | --- | --- | --- | --- | --- | --- | --- | --- | --- | --- |
| **UL6Af** | **-** | **-** | **-** | **-** | **-** | **-** | **-** | **-** | **-** | **-** | **-** | **-** | **-** | **-** | **-** | **-** | **-** | **-** | **-** | **UL6Af** |
| **UL6Am** | **-** | **-** | **-** | **-** | **-** | **-** | **-** | **-** | **-** | **-** | **-** | **-** | **-** | **-** | **-** | **-** | **-** | **-** | **-** | **UL6Am** |
| **UL6Bf** | **-** | **-** | **-** | **-** | **-** | **-** | **-** | **-** | **-** | **-** | **-** | **-** | **-** | **-** | **-** | **-** | **-** | **-** | **-** | **UL6Bf** |
| **UL6Bm** | **-** | **-** | **-** | **-** | **-** | **-** | **-** | **-** | **-** | **-** | **-** | **-** | **-** | **-** | **-** | **-** | **-** | **-** | **-** | **UL6Bm** |
| **UL6Cf** | **-** | **-** | **-** | **-** | **-** | **-** | **-** | **-** | **-** | **-** | **-** | **-** | **-** | **-** | **-** | **-** | **-** | **-** | **-** | **UL6Cf** |
| **UL6Cm** | **-** | **-** | **-** | **-** | **-** | **-** | **-** | **-** | **-** | **-** | **-** | **-** | **-** | **-** | **-** | **-** | **-** | **-** | **-** | **UL6Cm** |
| **UL6Df** | 127 | 3.64 | 0.94 | 0.08 | 3.48 | 2.13 | 6.76 | 4.63 | 3.42 | 3.85 | 2.16 | 2.34 | 2.56 | 2.91 | 3.48 | 4.22 | 4.77 | 5.12 | 6.47 | **UL6Df** |
| **UL6Dm** | 127 | 3.54 | 0.83 | 0.07 | 3.57 | 2.17 | 6.15 | 3.98 | 3.36 | 3.73 | 2.24 | 2.46 | 2.53 | 2.80 | 3.57 | 4.11 | 4.70 | 4.86 | 5.53 | **UL6Dm** |
| **UL6Ef** | 66 | 5.17 | 0.58 | 0.07 | 5.17 | 3.95 | 6.38 | 2.43 | 4.99 | 5.36 | 3.96 | 4.23 | 4.28 | 4.85 | 5.17 | 5.52 | 5.89 | 6.09 | 6.36 | **UL6Ef** |
| **UL6Em** | 86 | 5.25 | 0.74 | 0.08 | 5.22 | 2.97 | 7.27 | 4.30 | 5.05 | 5.46 | 3.09 | 3.96 | 4.42 | 5.00 | 5.22 | 5.61 | 6.09 | 6.50 | 7.07 | **UL6Em** |
| **UL6Ff** | 75 | 5.80 | 0.64 | 0.07 | 5.69 | 4.38 | 7.85 | 3.47 | 5.61 | 5.99 | 4.57 | 5.05 | 5.14 | 5.40 | 5.69 | 6.11 | 6.50 | 7.07 | 7.79 | **UL6Ff** |
| **UL6Fm** | 72 | 6.15 | 0.66 | 0.08 | 6.05 | 5.05 | 8.03 | 2.99 | 5.95 | 6.35 | 5.07 | 5.22 | 5.37 | 5.66 | 6.05 | 6.48 | 6.96 | 7.43 | 7.98 | **UL6Fm** |
| **UL6Gf** | 82 | 7.19 | 0.91 | 0.10 | 7.20 | 5.56 | 10.64 | 5.09 | 6.93 | 7.45 | 5.58 | 5.81 | 5.94 | 6.66 | 7.20 | 7.69 | 7.97 | 8.64 | 10.39 | **UL6Gf** |
| **UL6Gm** | 85 | 7.55 | 1.03 | 0.11 | 7.42 | 5.49 | 9.76 | 4.26 | 7.26 | 7.83 | 5.54 | 5.86 | 5.96 | 7.01 | 7.42 | 8.14 | 8.89 | 9.27 | 9.70 | **UL6Gm** |
| **UL6Hf** | 118 | 9.07 | 0.79 | 0.07 | 9.16 | 7.21 | 10.37 | 3.16 | 8.88 | 9.26 | 7.40 | 7.78 | 8.11 | 8.40 | 9.16 | 9.75 | 10.10 | 10.24 | 10.37 | **UL6Hf** |
| **UL6Hm** | 94 | 9.24 | 0.77 | 0.08 | 9.31 | 7.59 | 10.36 | 2.77 | 9.04 | 9.45 | 7.64 | 7.83 | 8.07 | 8.78 | 9.31 | 9.92 | 10.15 | 10.24 | 10.36 | **UL6Hm** |
| TDS | n | mean | sd | se | med | min | max | range | 99%CIL | 99%CIU | 0.5%ile | 5%ile | 10%ile | 25%ile | 50%ile | 75%ile | 90%ile | 95%ile | 99.5%ile | TDS |

**Table S1g.** Summary data of maxillary left second molar (UL7 f & m)

| TDS | n | mean | sd | se | med | min | max | range | 99%CIL | 99%CIU | 0.5%ile | 5%ile | 10%ile | 25%ile | 50%ile | 75%ile | 90%ile | 95%ile | 99.5%ile | TDS |
| --- | --- | --- | --- | --- | --- | --- | --- | --- | --- | --- | --- | --- | --- | --- | --- | --- | --- | --- | --- | --- |
| **UL7Af** | 25 | 4.15 | 0.52 | 0.10 | 3.98 | 3.32 | 5.17 | 1.85 | 3.88 | 4.41 | 3.32 | 3.39 | 3.62 | 3.79 | 3.98 | 4.52 | 4.97 | 5.08 | 5.16 | **UL7Af** |
| **UL7Am** | 30 | 4.21 | 0.45 | 0.08 | 4.14 | 3.33 | 5.12 | 1.79 | 4.00 | 4.42 | 3.37 | 3.66 | 3.74 | 3.87 | 4.14 | 4.59 | 4.77 | 4.95 | 5.11 | **UL7Am** |
| **UL7Bf** | 54 | 4.94 | 0.71 | 0.10 | 4.92 | 2.95 | 6.57 | 3.62 | 4.69 | 5.18 | 3.09 | 3.91 | 4.23 | 4.57 | 4.92 | 5.32 | 5.82 | 6.15 | 6.52 | **UL7Bf** |
| **UL7Bm** | 53 | 4.86 | 0.71 | 0.10 | 4.86 | 3.41 | 6.37 | 2.96 | 4.61 | 5.11 | 3.43 | 3.70 | 3.82 | 4.38 | 4.86 | 5.21 | 5.72 | 6.14 | 6.34 | **UL7Bm** |
| **UL7Cf** | 78 | 5.69 | 0.71 | 0.08 | 5.57 | 4.30 | 8.56 | 4.27 | 5.48 | 5.89 | 4.33 | 4.99 | 5.06 | 5.25 | 5.57 | 5.96 | 6.32 | 7.05 | 8.37 | **UL7Cf** |
| **UL7Cm** | 105 | 5.74 | 0.68 | 0.07 | 5.62 | 3.47 | 8.15 | 4.68 | 5.57 | 5.91 | 3.96 | 4.94 | 5.08 | 5.32 | 5.62 | 6.05 | 6.58 | 7.02 | 7.74 | **UL7Cm** |
| **UL7Df** | 186 | 7.69 | 1.27 | 0.09 | 7.70 | 5.13 | 10.37 | 5.24 | 7.45 | 7.93 | 5.15 | 5.62 | 5.86 | 6.75 | 7.70 | 8.58 | 9.35 | 9.78 | 10.31 | **UL7Df** |
| **UL7Dm** | 175 | 8.09 | 1.27 | 0.10 | 8.09 | 5.45 | 10.82 | 5.38 | 7.85 | 8.34 | 5.63 | 6.01 | 6.39 | 7.24 | 8.09 | 9.09 | 9.89 | 10.11 | 10.65 | **UL7Dm** |
| **UL7Ef** | 64 | 10.51 | 0.94 | 0.12 | 10.46 | 8.40 | 12.83 | 4.43 | 10.21 | 10.81 | 8.57 | 9.15 | 9.39 | 9.82 | 10.46 | 11.12 | 11.80 | 11.99 | 12.81 | **UL7Ef** |
| **UL7Em** | 71 | 10.84 | 1.11 | 0.13 | 10.72 | 8.70 | 13.89 | 5.20 | 10.50 | 11.18 | 8.83 | 9.21 | 9.56 | 10.14 | 10.72 | 11.57 | 12.13 | 12.79 | 13.83 | **UL7Em** |
| **UL7Ff** | 51 | 11.69 | 1.02 | 0.14 | 11.86 | 8.85 | 13.84 | 4.99 | 11.33 | 12.06 | 9.06 | 9.94 | 10.57 | 10.92 | 11.86 | 12.52 | 12.82 | 12.97 | 13.64 | **UL7Ff** |
| **UL7Fm** | 42 | 11.69 | 0.98 | 0.15 | 11.53 | 9.69 | 14.86 | 5.17 | 11.30 | 12.08 | 9.79 | 10.49 | 10.62 | 11.15 | 11.53 | 12.06 | 13.06 | 13.16 | 14.68 | **UL7Fm** |
| **UL7Gf** | 94 | 13.25 | 1.46 | 0.15 | 13.22 | 10.23 | 17.00 | 6.77 | 12.86 | 13.64 | 10.27 | 10.68 | 11.40 | 12.37 | 13.22 | 14.17 | 14.85 | 15.46 | 16.88 | **UL7Gf** |
| **UL7Gm** | 93 | 13.21 | 1.27 | 0.13 | 13.20 | 10.89 | 17.02 | 6.12 | 12.87 | 13.55 | 10.98 | 11.40 | 11.58 | 12.31 | 13.20 | 13.91 | 14.81 | 15.48 | 16.81 | **UL7Gm** |
| **UL7Hf** | 81 | 14.17 | 0.96 | 0.11 | 14.28 | 11.73 | 15.50 | 3.78 | 13.90 | 14.45 | 11.76 | 12.33 | 13.05 | 13.47 | 14.28 | 14.98 | 15.30 | 15.38 | 15.48 | **UL7Hf** |
| **UL7Hm** | 116 | 15.12 | 1.07 | 0.10 | 15.20 | 12.05 | 16.72 | 4.67 | 14.87 | 15.38 | 12.13 | 13.31 | 13.89 | 14.32 | 15.20 | 15.94 | 16.48 | 16.66 | 16.71 | **UL7Hm** |
| TDS | n | mean | sd | se | med | min | max | range | 99%CIL | 99%CIU | 0.5%ile | 5%ile | 10%ile | 25%ile | 50%ile | 75%ile | 90%ile | 95%ile | 99.5%ile | TDS |

**Table S1h.** Summary data of mandibular left second molar (LL7 f & m)

| TDS | n | mean | sd | se | med | min | max | range | 99%CIL | 99%CIU | 0.5%ile | 5%ile | 10%ile | 25%ile | 50%ile | 75%ile | 90%ile | 95%ile | 99.5%ile | TDS |
| --- | --- | --- | --- | --- | --- | --- | --- | --- | --- | --- | --- | --- | --- | --- | --- | --- | --- | --- | --- | --- |
| **LL7Af** | 37 | 4.09 | 0.49 | 0.08 | 4.02 | 2.90 | 5.17 | 2.27 | 3.88 | 4.30 | 2.98 | 3.34 | 3.58 | 3.79 | 4.02 | 4.44 | 4.67 | 4.95 | 5.14 | **LL7Af** |
| **LL7Am** | 43 | 4.18 | 0.54 | 0.08 | 4.03 | 3.33 | 6.13 | 2.80 | 3.97 | 4.40 | 3.38 | 3.59 | 3.63 | 3.82 | 4.03 | 4.52 | 4.77 | 5.12 | 5.93 | **LL7Am** |
| **LL7Bf** | 56 | 4.88 | 0.73 | 0.10 | 4.92 | 2.95 | 6.57 | 3.62 | 4.63 | 5.13 | 3.01 | 3.70 | 4.10 | 4.49 | 4.92 | 5.30 | 5.68 | 6.15 | 6.52 | **LL7Bf** |
| **LL7Bm** | 62 | 4.95 | 0.67 | 0.09 | 5.06 | 3.41 | 6.37 | 2.96 | 4.73 | 5.17 | 3.44 | 3.67 | 4.09 | 4.57 | 5.06 | 5.37 | 5.76 | 6.00 | 6.34 | **LL7Bm** |
| **LL7Cf** | 94 | 5.84 | 0.78 | 0.08 | 5.71 | 4.30 | 8.38 | 4.09 | 5.64 | 6.05 | 4.33 | 5.03 | 5.09 | 5.30 | 5.71 | 6.11 | 6.75 | 7.59 | 8.29 | **LL7Cf** |
| **LL7Cm** | 115 | 6.06 | 0.92 | 0.09 | 5.91 | 3.47 | 8.72 | 5.25 | 5.84 | 6.29 | 4.01 | 4.99 | 5.10 | 5.44 | 5.91 | 6.54 | 7.36 | 8.04 | 8.58 | **LL7Cm** |
| **LL7Df** | 156 | 7.68 | 1.22 | 0.10 | 7.70 | 5.13 | 10.37 | 5.24 | 7.43 | 7.93 | 5.15 | 5.66 | 5.89 | 6.84 | 7.70 | 8.56 | 9.27 | 9.51 | 10.24 | **LL7Df** |
| **LL7Dm** | 141 | 8.01 | 1.25 | 0.11 | 8.06 | 5.10 | 10.62 | 5.52 | 7.74 | 8.29 | 5.34 | 5.90 | 6.31 | 7.26 | 8.06 | 8.90 | 9.65 | 9.99 | 10.55 | **LL7Dm** |
| **LL7Ef** | 75 | 10.20 | 1.16 | 0.13 | 10.10 | 7.57 | 13.07 | 5.49 | 9.85 | 10.54 | 7.59 | 8.32 | 8.78 | 9.58 | 10.10 | 10.86 | 11.74 | 12.07 | 13.02 | **LL7Ef** |
| **LL7Em** | 80 | 10.52 | 1.05 | 0.12 | 10.46 | 8.70 | 13.13 | 4.43 | 10.21 | 10.82 | 8.77 | 9.04 | 9.15 | 9.66 | 10.46 | 11.17 | 11.99 | 12.52 | 13.04 | **LL7Em** |
| **LL7Ff** | 54 | 11.65 | 0.94 | 0.13 | 11.69 | 9.70 | 13.84 | 4.14 | 11.32 | 11.98 | 9.76 | 9.97 | 10.59 | 10.89 | 11.69 | 12.43 | 12.77 | 12.98 | 13.63 | **LL7Ff** |
| **LL7Fm** | 51 | 11.75 | 1.00 | 0.14 | 11.72 | 9.69 | 14.86 | 5.17 | 11.39 | 12.11 | 9.81 | 10.34 | 10.61 | 11.16 | 11.72 | 12.13 | 13.07 | 13.53 | 14.64 | **LL7Fm** |
| **LL7Gf** | 106 | 13.26 | 1.51 | 0.15 | 13.24 | 8.85 | 17.35 | 8.50 | 12.88 | 13.64 | 9.58 | 10.62 | 11.44 | 12.42 | 13.24 | 14.19 | 14.97 | 15.63 | 17.04 | **LL7Gf** |
| **LL7Gm** | 101 | 13.36 | 1.36 | 0.14 | 13.31 | 10.89 | 17.60 | 6.70 | 13.01 | 13.71 | 10.99 | 11.42 | 11.58 | 12.35 | 13.31 | 14.11 | 15.33 | 15.94 | 17.09 | **LL7Gm** |
| **LL7Hf** | 127 | 15.13 | 1.21 | 0.11 | 15.30 | 12.32 | 16.88 | 4.56 | 14.85 | 15.40 | 12.33 | 13.12 | 13.34 | 14.22 | 15.30 | 16.16 | 16.58 | 16.76 | 16.88 | **LL7Hf** |
| **LL7Hm** | 116 | 15.12 | 1.07 | 0.10 | 15.20 | 12.05 | 16.72 | 4.67 | 14.87 | 15.38 | 12.13 | 13.31 | 13.89 | 14.32 | 15.20 | 15.94 | 16.48 | 16.66 | 16.71 | **LL7Hm** |
| TDS | n | mean | sd | se | med | min | max | range | 99%CIL | 99%CIU | 0.5%ile | 5%ile | 10%ile | 25%ile | 50%ile | 75%ile | 90%ile | 95%ile | 99.5%ile | TDS |

**Table S1i.** Summary data of mandibular left first molar (LL6 f & m)

| TDS | n | mean | sd | se | med | min | max | range | 99%CIL | 99%CIU | 0.5%ile | 5%ile | 10%ile | 25%ile | 50%ile | 75%ile | 90%ile | 95%ile | 99.5%ile | TDS |
| --- | --- | --- | --- | --- | --- | --- | --- | --- | --- | --- | --- | --- | --- | --- | --- | --- | --- | --- | --- | --- |
| **LL6Af** | **-** | **-** | **-** | **-** | **-** | **-** | **-** | **-** | **-** | **-** | **-** | **-** | **-** | **-** | **-** | **-** | **-** | **-** | **-** | **LL6Af** |
| **LL6Am** | **-** | **-** | **-** | **-** | **-** | **-** | **-** | **-** | **-** | **-** | **-** | **-** | **-** | **-** | **-** | **-** | **-** | **-** | **-** | **LL6Am** |
| **LL6Bf** | **-** | **-** | **-** | **-** | **-** | **-** | **-** | **-** | **-** | **-** | **-** | **-** | **-** | **-** | **-** | **-** | **-** | **-** | **-** | **LL6Bf** |
| **LL6Bm** | **-** | **-** | **-** | **-** | **-** | **-** | **-** | **-** | **-** | **-** | **-** | **-** | **-** | **-** | **-** | **-** | **-** | **-** | **-** | **LL6Bm** |
| **LL6Cf** | 14 | 2.41 | 0.21 | 0.06 | 2.41 | 2.15 | 2.94 | 0.80 | 2.27 | 2.56 | 2.15 | 2.17 | 2.18 | 2.29 | 2.41 | 2.45 | 2.63 | 2.79 | 2.93 | **LL6Cf** |
| **LL6Cm** | 9 | 2.48 | 0.19 | 0.06 | 2.50 | 2.17 | 2.77 | 0.61 | 2.32 | 2.64 | 2.17 | 2.21 | 2.26 | 2.40 | 2.50 | 2.54 | 2.70 | 2.74 | 2.77 | **LL6Cm** |
| **LL6Df** | 102 | 3.39 | 0.68 | 0.07 | 3.36 | 2.13 | 4.90 | 2.77 | 3.21 | 3.56 | 2.15 | 2.34 | 2.47 | 2.87 | 3.36 | 3.84 | 4.30 | 4.61 | 4.87 | **LL6Df** |
| **LL6Dm** | 104 | 3.51 | 0.82 | 0.08 | 3.48 | 2.17 | 6.15 | 3.98 | 3.30 | 3.71 | 2.26 | 2.50 | 2.55 | 2.80 | 3.48 | 4.01 | 4.65 | 4.91 | 5.64 | **LL6Dm** |
| **LL6Ef** | 82 | 5.06 | 0.71 | 0.08 | 5.07 | 2.90 | 6.76 | 3.85 | 4.85 | 5.26 | 3.04 | 4.07 | 4.23 | 4.60 | 5.07 | 5.48 | 6.08 | 6.19 | 6.60 | **LL6Ef** |
| **LL6Em** | 99 | 5.10 | 0.82 | 0.08 | 5.12 | 3.24 | 7.27 | 4.02 | 4.88 | 5.31 | 3.24 | 3.71 | 3.92 | 4.66 | 5.12 | 5.52 | 6.04 | 6.54 | 7.21 | **LL6Em** |
| **LL6Ff** | 82 | 5.79 | 0.60 | 0.07 | 5.69 | 4.38 | 7.70 | 3.33 | 5.62 | 5.96 | 4.59 | 5.06 | 5.14 | 5.42 | 5.69 | 6.09 | 6.51 | 7.03 | 7.65 | **LL6Ff** |
| **LL6Fm** | 78 | 6.12 | 0.67 | 0.08 | 6.04 | 5.05 | 8.03 | 2.99 | 5.93 | 6.32 | 5.07 | 5.25 | 5.36 | 5.61 | 6.04 | 6.46 | 7.02 | 7.40 | 7.97 | **LL6Fm** |
| **LL6Gf** | 94 | 7.42 | 1.05 | 0.11 | 7.39 | 5.56 | 11.68 | 6.12 | 7.14 | 7.70 | 5.58 | 5.85 | 6.07 | 6.75 | 7.39 | 7.86 | 8.69 | 9.35 | 10.90 | **LL6Gf** |
| **LL6Gm** | 95 | 7.70 | 1.15 | 0.12 | 7.81 | 5.49 | 12.71 | 7.21 | 7.40 | 8.01 | 5.55 | 5.88 | 6.01 | 7.06 | 7.81 | 8.21 | 8.90 | 9.34 | 11.45 | **LL6Gm** |
| **LL6Hf** | 195 | 10.18 | 1.33 | 0.10 | 10.20 | 7.53 | 12.37 | 4.85 | 9.93 | 10.42 | 7.58 | 8.11 | 8.38 | 9.13 | 10.20 | 11.38 | 11.92 | 12.19 | 12.37 | **LL6Hf** |
| **LL6Hm** | 165 | 10.24 | 1.11 | 0.09 | 10.35 | 7.59 | 11.90 | 4.31 | 10.02 | 10.47 | 7.68 | 8.22 | 8.73 | 9.40 | 10.35 | 11.17 | 11.62 | 11.75 | 11.89 | **LL6Hm** |
| TDS | n | mean | sd | se | med | min | max | range | 99%CIL | 99%CIU | 0.5%ile | 5%ile | 10%ile | 25%ile | 50%ile | 75%ile | 90%ile | 95%ile | 99.5%ile | TDS |

**Table S1j.** Summary data of mandibular left second pre-molar (LL5 f & m)

| TDS | n | mean | sd | se | med | min | max | range | 99%CIL | 99%CIU | 0.5%ile | 5%ile | 10%ile | 25%ile | 50%ile | 75%ile | 90%ile | 95%ile | 99.5%ile | TDS |
| --- | --- | --- | --- | --- | --- | --- | --- | --- | --- | --- | --- | --- | --- | --- | --- | --- | --- | --- | --- | --- |
| **LL5Af** | 26 | 3.81 | 0.66 | 0.13 | 3.78 | 2.86 | 5.74 | 2.88 | 3.48 | 4.15 | 2.87 | 2.91 | 3.03 | 3.40 | 3.78 | 4.01 | 4.43 | 5.09 | 5.68 | **LL5Af** |
| **LL5Am** | 29 | 4.00 | 0.67 | 0.12 | 3.92 | 2.96 | 5.40 | 2.44 | 3.68 | 4.33 | 2.96 | 2.97 | 2.97 | 3.70 | 3.92 | 4.49 | 5.10 | 5.15 | 5.37 | **LL5Am** |
| **LL5Bf** | 68 | 4.66 | 0.67 | 0.08 | 4.67 | 3.16 | 6.38 | 3.22 | 4.45 | 4.87 | 3.20 | 3.63 | 3.79 | 4.22 | 4.67 | 5.08 | 5.52 | 5.68 | 6.30 | **LL5Bf** |
| **LL5Bm** | 62 | 4.58 | 0.75 | 0.09 | 4.62 | 3.24 | 6.96 | 3.72 | 4.34 | 4.83 | 3.24 | 3.57 | 3.67 | 4.01 | 4.62 | 5.08 | 5.47 | 5.84 | 6.71 | **LL5Bm** |
| **LL5Cf** | 71 | 5.65 | 0.92 | 0.11 | 5.49 | 2.95 | 8.56 | 5.61 | 5.37 | 5.93 | 3.40 | 4.35 | 4.90 | 5.09 | 5.49 | 6.09 | 6.74 | 7.35 | 8.43 | **LL5Cf** |
| **LL5Cm** | 76 | 5.68 | 0.71 | 0.08 | 5.53 | 3.47 | 8.15 | 4.68 | 5.47 | 5.89 | 3.93 | 4.82 | 4.96 | 5.18 | 5.53 | 6.11 | 6.54 | 6.89 | 7.86 | **LL5Cm** |
| **LL5Df** | 129 | 6.86 | 1.14 | 0.10 | 6.75 | 5.16 | 10.37 | 5.22 | 6.60 | 7.12 | 5.16 | 5.42 | 5.54 | 5.83 | 6.75 | 7.69 | 8.38 | 8.69 | 10.12 | **LL5Df** |
| **LL5Dm** | 133 | 7.01 | 1.20 | 0.10 | 6.99 | 4.42 | 10.06 | 5.65 | 6.74 | 7.28 | 4.80 | 5.33 | 5.60 | 5.97 | 6.99 | 7.87 | 8.81 | 9.16 | 9.78 | **LL5Dm** |
| **LL5Ef** | 64 | 8.61 | 0.81 | 0.10 | 8.54 | 7.21 | 10.64 | 3.43 | 8.34 | 8.87 | 7.26 | 7.47 | 7.56 | 8.00 | 8.54 | 9.22 | 9.56 | 9.99 | 10.51 | **LL5Ef** |
| **LL5Em** | 59 | 8.82 | 1.12 | 0.15 | 8.78 | 6.48 | 12.54 | 6.06 | 8.44 | 9.19 | 6.52 | 7.16 | 7.70 | 8.07 | 8.78 | 9.43 | 10.06 | 10.53 | 12.17 | **LL5Em** |
| **LL5Ff** | 113 | 10.97 | 1.27 | 0.12 | 10.82 | 7.63 | 14.02 | 6.39 | 10.66 | 11.28 | 8.21 | 9.12 | 9.39 | 9.99 | 10.82 | 11.95 | 12.68 | 13.03 | 13.92 | **LL5Ff** |
| **LL5Fm** | 126 | 11.00 | 1.27 | 0.11 | 10.89 | 8.20 | 14.86 | 6.66 | 10.71 | 11.30 | 8.39 | 9.11 | 9.51 | 10.12 | 10.89 | 11.84 | 12.77 | 13.18 | 14.31 | **LL5Fm** |
| **LL5Gf** | 75 | 12.69 | 1.48 | 0.17 | 12.75 | 8.85 | 16.77 | 7.93 | 12.25 | 13.13 | 9.12 | 10.33 | 10.85 | 11.61 | 12.75 | 13.64 | 14.36 | 14.80 | 16.60 | **LL5Gf** |
| **LL5Gm** | 71 | 12.70 | 1.26 | 0.15 | 12.56 | 10.74 | 16.41 | 5.67 | 12.31 | 13.09 | 10.79 | 11.01 | 11.22 | 11.77 | 12.56 | 13.33 | 14.27 | 15.13 | 16.24 | **LL5Gm** |
| **LL5Hf** | 124 | 14.22 | 1.22 | 0.11 | 14.44 | 11.13 | 15.96 | 4.84 | 13.94 | 14.50 | 11.28 | 11.86 | 12.49 | 13.42 | 14.44 | 15.21 | 15.62 | 15.81 | 15.91 | **LL5Hf** |
| **LL5Hm** | 168 | 15.13 | 1.24 | 0.10 | 15.15 | 12.20 | 17.16 | 4.97 | 14.88 | 15.38 | 12.30 | 13.04 | 13.52 | 14.18 | 15.15 | 16.16 | 16.78 | 16.95 | 17.16 | **LL5Hm** |
| TDS | n | mean | sd | se | med | min | max | range | 99%CIL | 99%CIU | 0.5%ile | 5%ile | 10%ile | 25%ile | 50%ile | 75%ile | 90%ile | 95%ile | 99.5%ile | TDS |

**Table S1k.** Summary data of mandibular left first pre-molar (LL4 f & m)

| TDS | n | mean | sd | se | med | min | max | range | 99%CIL | 99%CIU | 0.5%ile | 5%ile | 10%ile | 25%ile | 50%ile | 75%ile | 90%ile | 95%ile | 99.5%ile | TDS |
| --- | --- | --- | --- | --- | --- | --- | --- | --- | --- | --- | --- | --- | --- | --- | --- | --- | --- | --- | --- | --- |
| **LL4Af** | 24 | 2.91 | 0.40 | 0.08 | 2.88 | 2.33 | 4.30 | 1.97 | 2.70 | 3.12 | 2.34 | 2.44 | 2.46 | 2.72 | 2.88 | 2.98 | 3.28 | 3.41 | 4.20 | **LL4Af** |
| **LL4Am** | 26 | 2.91 | 0.46 | 0.09 | 2.85 | 2.43 | 4.55 | 2.12 | 2.68 | 3.14 | 2.44 | 2.50 | 2.53 | 2.59 | 2.85 | 2.98 | 3.31 | 3.73 | 4.46 | **LL4Am** |
| **LL4Bf** | 49 | 3.73 | 0.68 | 0.10 | 3.59 | 2.75 | 6.18 | 3.43 | 3.48 | 3.99 | 2.77 | 2.88 | 2.94 | 3.25 | 3.59 | 4.07 | 4.65 | 4.87 | 5.88 | **LL4Bf** |
| **LL4Bm** | 60 | 3.87 | 0.58 | 0.07 | 3.84 | 2.67 | 4.96 | 2.30 | 3.67 | 4.06 | 2.67 | 2.87 | 2.97 | 3.54 | 3.84 | 4.25 | 4.67 | 4.78 | 4.92 | **LL4Bm** |
| **LL4Cf** | 71 | 4.49 | 0.64 | 0.08 | 4.57 | 2.95 | 5.85 | 2.90 | 4.30 | 4.69 | 3.02 | 3.39 | 3.67 | 4.00 | 4.57 | 4.98 | 5.28 | 5.39 | 5.79 | **LL4Cf** |
| **LL4Cm** | 75 | 4.92 | 0.76 | 0.09 | 5.07 | 3.24 | 6.60 | 3.36 | 4.69 | 5.14 | 3.31 | 3.58 | 3.81 | 4.54 | 5.07 | 5.40 | 5.88 | 6.08 | 6.58 | **LL4Cm** |
| **LL4Df** | 161 | 6.17 | 0.97 | 0.08 | 5.90 | 4.30 | 10.37 | 6.08 | 5.97 | 6.36 | 4.68 | 5.09 | 5.17 | 5.51 | 5.90 | 6.62 | 7.55 | 8.05 | 9.81 | **LL4Df** |
| **LL4Dm** | 154 | 6.40 | 1.00 | 0.08 | 6.21 | 4.42 | 9.62 | 5.20 | 6.19 | 6.61 | 4.86 | 5.11 | 5.32 | 5.64 | 6.21 | 7.15 | 7.74 | 8.12 | 9.44 | **LL4Dm** |
| **LL4Ef** | 76 | 8.13 | 0.85 | 0.10 | 8.15 | 5.90 | 10.06 | 4.16 | 7.88 | 8.38 | 5.91 | 6.75 | 7.20 | 7.54 | 8.15 | 8.57 | 9.20 | 9.54 | 10.04 | **LL4Ef** |
| **LL4Em** | 77 | 8.37 | 0.93 | 0.11 | 8.28 | 6.27 | 10.62 | 4.36 | 8.09 | 8.64 | 6.28 | 6.60 | 7.12 | 7.85 | 8.28 | 8.90 | 9.36 | 9.91 | 10.48 | **LL4Em** |
| **LL4Ff** | 107 | 10.21 | 1.41 | 0.14 | 10.08 | 7.37 | 13.84 | 6.47 | 9.86 | 10.57 | 7.42 | 7.82 | 8.57 | 9.31 | 10.08 | 10.92 | 12.26 | 12.90 | 13.50 | **LL4Ff** |
| **LL4Fm** | 112 | 10.66 | 1.27 | 0.12 | 10.46 | 8.20 | 14.86 | 6.66 | 10.35 | 10.97 | 8.37 | 9.01 | 9.17 | 9.67 | 10.46 | 11.56 | 12.50 | 13.07 | 14.37 | **LL4Fm** |
| **LL4Gf** | 76 | 12.12 | 1.32 | 0.15 | 12.14 | 9.59 | 14.87 | 5.28 | 11.73 | 12.51 | 9.67 | 10.11 | 10.40 | 10.94 | 12.14 | 12.84 | 14.00 | 14.74 | 14.85 | **LL4Gf** |
| **LL4Gm** | 80 | 12.30 | 1.25 | 0.14 | 12.05 | 9.88 | 16.41 | 6.53 | 11.94 | 12.66 | 10.10 | 10.65 | 11.05 | 11.37 | 12.05 | 13.10 | 13.75 | 14.49 | 16.14 | **LL4Gm** |
| **LL4Hf** | 86 | 13.05 | 0.92 | 0.10 | 13.30 | 10.72 | 14.27 | 3.55 | 12.80 | 13.31 | 10.78 | 11.39 | 11.63 | 12.48 | 13.30 | 13.76 | 14.08 | 14.18 | 14.25 | **LL4Hf** |
| **LL4Hm** | 66 | 13.29 | 0.81 | 0.10 | 13.52 | 10.89 | 14.21 | 3.32 | 13.04 | 13.55 | 10.90 | 11.77 | 12.19 | 12.75 | 13.52 | 13.96 | 14.08 | 14.18 | 14.21 | **LL4Hm** |
| TDS | n | mean | sd | se | med | min | max | range | 99%CIL | 99%CIU | 0.5%ile | 5%ile | 10%ile | 25%ile | 50%ile | 75%ile | 90%ile | 95%ile | 99.5%ile | TDS |

**Table S1l.** Summary data of mandibular left canine (LL3 f & m)

| TDS | n | mean | sd | se | med | min | max | range | 99%CIL | 99%CIU | 0.5%ile | 5%ile | 10%ile | 25%ile | 50%ile | 75%ile | 90%ile | 95%ile | 99.5%ile | TDS |
| --- | --- | --- | --- | --- | --- | --- | --- | --- | --- | --- | --- | --- | --- | --- | --- | --- | --- | --- | --- | --- |
| **LL3Af** | **-** | **-** | **-** | **-** | **-** | **-** | **-** | **-** | **-** | **-** | **-** | **-** | **-** | **-** | **-** | **-** | **-** | **-** | **-** | **LL3Af** |
| **LL3Am** | **-** | **-** | **-** | **-** | **-** | **-** | **-** | **-** | **-** | **-** | **-** | **-** | **-** | **-** | **-** | **-** | **-** | **-** | **-** | **LL3Am** |
| **LL3Bf** | 2 | 2.26 | 0.15 | 0.11 | 2.26 | 2.15 | 2.37 | 0.22 | 1.97 | 2.54 | 2.15 | 2.16 | 2.17 | 2.37 | 2.26 | 2.31 | 2.34 | 2.35 | 2.36 | **LL3Bf** |
| **LL3Bm** | 2 | 2.52 | 0.03 | 0.02 | 2.52 | 2.50 | 2.54 | 0.04 | 2.47 | 2.57 | 2.50 | 2.50 | 2.50 | 2.50 | 2.52 | 2.53 | 2.53 | 2.54 | 2.54 | **LL3Bm** |
| **LL3Cf** | 78 | 3.06 | 0.65 | 0.07 | 2.92 | 2.13 | 4.83 | 2.70 | 2.87 | 3.26 | 2.15 | 2.24 | 2.32 | 2.63 | 2.92 | 3.42 | 4.02 | 4.29 | 4.78 | **LL3Cf** |
| **LL3Cm** | 76 | 3.18 | 0.75 | 0.09 | 2.92 | 2.17 | 6.15 | 3.98 | 2.95 | 3.40 | 2.21 | 2.42 | 2.50 | 2.67 | 2.92 | 3.74 | 4.06 | 4.66 | 5.78 | **LL3Cm** |
| **LL3Df** | 175 | 5.04 | 0.98 | 0.07 | 5.10 | 2.77 | 7.70 | 4.93 | 4.85 | 5.23 | 2.88 | 3.30 | 3.78 | 4.31 | 5.10 | 5.69 | 6.19 | 6.48 | 7.69 | **LL3Df** |
| **LL3Dm** | 210 | 5.35 | 1.06 | 0.07 | 5.38 | 2.52 | 8.11 | 5.59 | 5.16 | 5.54 | 2.98 | 3.59 | 3.86 | 4.69 | 5.38 | 6.00 | 6.79 | 7.20 | 8.02 | **LL3Dm** |
| **LL3Ef** | 103 | 6.86 | 1.05 | 0.10 | 6.87 | 4.90 | 10.01 | 5.11 | 6.60 | 7.13 | 5.08 | 5.40 | 5.55 | 5.91 | 6.87 | 7.69 | 8.23 | 8.40 | 9.51 | **LL3Ef** |
| **LL3Em** | 87 | 7.53 | 1.10 | 0.12 | 7.66 | 5.37 | 10.06 | 4.70 | 7.22 | 7.83 | 5.40 | 5.89 | 5.98 | 6.62 | 7.66 | 8.16 | 8.86 | 9.18 | 9.99 | **LL3Em** |
| **LL3Ff** | 101 | 8.87 | 0.96 | 0.10 | 8.79 | 6.75 | 10.91 | 4.15 | 8.62 | 9.11 | 6.98 | 7.47 | 7.57 | 8.10 | 8.79 | 9.51 | 10.11 | 10.50 | 10.88 | **LL3Ff** |
| **LL3Fm** | 157 | 10.15 | 1.36 | 0.11 | 10.14 | 6.62 | 13.13 | 6.51 | 9.87 | 10.43 | 6.91 | 7.96 | 8.26 | 9.21 | 10.14 | 11.15 | 12.00 | 12.34 | 13.09 | **LL3Fm** |
| **LL3Gf** | 110 | 11.71 | 1.44 | 0.14 | 11.75 | 9.05 | 15.29 | 6.24 | 11.36 | 12.07 | 9.09 | 9.64 | 9.82 | 10.60 | 11.75 | 12.62 | 13.56 | 14.44 | 15.17 | **LL3Gf** |
| **LL3Gm** | 81 | 12.75 | 1.47 | 0.16 | 12.71 | 10.18 | 17.73 | 7.55 | 12.33 | 13.17 | 10.43 | 10.92 | 11.09 | 11.75 | 12.71 | 13.38 | 14.47 | 15.58 | 17.67 | **LL3Gm** |
| **LL3Hf** | 96 | 12.73 | 1.00 | 0.10 | 13.04 | 10.27 | 14.06 | 3.79 | 12.47 | 13.00 | 10.39 | 10.81 | 11.20 | 11.93 | 13.04 | 13.54 | 13.84 | 13.95 | 14.04 | **LL3Hf** |
| **LL3Hm** | 28 | 12.65 | 0.77 | 0.15 | 12.82 | 10.74 | 13.66 | 2.92 | 12.27 | 13.03 | 10.82 | 11.42 | 11.56 | 12.19 | 12.82 | 13.33 | 13.57 | 13.62 | 13.66 | **LL3Hm** |
| TDS | n | mean | sd | se | med | min | max | range | 99%CIL | 99%CIU | 0.5%ile | 5%ile | 10%ile | 25%ile | 50%ile | 75%ile | 90%ile | 95%ile | 99.5%ile | TDS |

**Table S1m.** Summary data of mandibular left lateral incisor (LL2 f & m)

| TDS | n | mean | sd | se | med | min | max | range | 99%CIL | 99%CIU | 0.5%ile | 5%ile | 10%ile | 25%ile | 50%ile | 75%ile | 90%ile | 95%ile | 99.5%ile | TDS |
| --- | --- | --- | --- | --- | --- | --- | --- | --- | --- | --- | --- | --- | --- | --- | --- | --- | --- | --- | --- | --- |
| **LL2Af** | **-** | **-** | **-** | **-** | **-** | **-** | **-** | **-** | **-** | **-** | **-** | **-** | **-** | **-** | **-** | **-** | **-** | **-** | **-** | **LL2Af** |
| **LL2Am** | **-** | **-** | **-** | **-** | **-** | **-** | **-** | **-** | **-** | **-** | **-** | **-** | **-** | **-** | **-** | **-** | **-** | **-** | **-** | **LL2Am** |
| **LL2Bf** | **-** | **-** | **-** | **-** | **-** | **-** | **-** | **-** | **-** | **-** | **-** | **-** | **-** | **-** | **-** | **-** | **-** | **-** | **-** | **LL2Bf** |
| **LL2Bm** | **-** | **-** | **-** | **-** | **-** | **-** | **-** | **-** | **-** | **-** | **-** | **-** | **-** | **-** | **-** | **-** | **-** | **-** | **-** | **LL2Bm** |
| **LL2Cf** | 27 | 2.54 | 0.31 | 0.06 | 2.44 | 2.13 | 3.44 | 1.31 | 2.39 | 2.69 | 2.13 | 2.15 | 2.22 | 2.31 | 2.44 | 2.78 | 2.87 | 2.93 | 3.37 | **LL2Cf** |
| **LL2Cm** | 29 | 2.95 | 0.46 | 0.08 | 2.80 | 2.43 | 3.97 | 1.54 | 2.73 | 3.17 | 2.44 | 2.49 | 2.51 | 2.65 | 2.80 | 2.98 | 3.75 | 3.83 | 3.96 | **LL2Cm** |
| **LL2Df** | 128 | 3.89 | 0.84 | 0.07 | 3.88 | 2.18 | 6.18 | 4.00 | 3.70 | 4.09 | 2.19 | 2.67 | 2.89 | 3.24 | 3.88 | 4.53 | 4.95 | 5.20 | 6.11 | **LL2Df** |
| **LL2Dm** | 125 | 4.05 | 1.00 | 0.09 | 4.08 | 2.17 | 6.96 | 4.79 | 3.82 | 4.28 | 2.24 | 2.51 | 2.61 | 3.32 | 4.08 | 4.71 | 5.21 | 5.54 | 6.70 | **LL2Dm** |
| **LL2Ef** | 99 | 5.56 | 0.54 | 0.05 | 5.54 | 3.67 | 7.14 | 3.47 | 5.42 | 5.70 | 4.01 | 4.89 | 4.99 | 5.19 | 5.54 | 5.88 | 6.21 | 6.38 | 7.09 | **LL2Ef** |
| **LL2Em** | 102 | 5.74 | 0.56 | 0.06 | 5.66 | 3.92 | 7.15 | 3.23 | 5.59 | 5.88 | 4.39 | 5.05 | 5.10 | 5.33 | 5.66 | 6.12 | 6.48 | 6.74 | 7.11 | **LL2Em** |
| **LL2Ff** | 82 | 6.80 | 0.96 | 0.11 | 6.75 | 4.85 | 10.37 | 5.52 | 6.53 | 7.08 | 4.98 | 5.37 | 5.60 | 6.14 | 6.75 | 7.53 | 7.84 | 8.17 | 9.67 | **LL2Ff** |
| **LL2Fm** | 70 | 7.04 | 1.01 | 0.12 | 7.08 | 4.42 | 8.92 | 4.50 | 6.72 | 7.35 | 4.77 | 5.64 | 5.85 | 6.27 | 7.08 | 7.84 | 8.41 | 8.79 | 8.91 | **LL2Fm** |
| **LL2Gf** | 74 | 8.44 | 1.08 | 0.13 | 8.22 | 5.90 | 11.78 | 5.88 | 8.11 | 8.76 | 6.21 | 6.93 | 7.36 | 7.70 | 8.22 | 9.13 | 9.94 | 10.28 | 11.47 | **LL2Gf** |
| **LL2Gm** | 79 | 8.65 | 1.15 | 0.13 | 8.39 | 6.48 | 12.28 | 5.80 | 8.31 | 8.98 | 6.54 | 7.22 | 7.39 | 7.84 | 8.39 | 9.36 | 9.97 | 10.35 | 12.21 | **LL2Gm** |
| **LL2Hf** | 74 | 9.44 | 0.76 | 0.09 | 9.55 | 7.21 | 10.57 | 3.36 | 9.21 | 9.67 | 7.42 | 8.20 | 8.42 | 8.93 | 9.55 | 10.04 | 10.35 | 10.45 | 10.55 | **LL2Hf** |
| **LL2Hm** | 115 | 10.33 | 0.93 | 0.09 | 10.48 | 7.73 | 11.67 | 3.95 | 10.10 | 10.55 | 7.75 | 8.80 | 9.07 | 9.65 | 10.48 | 11.08 | 11.40 | 11.54 | 11.64 | **LL2Hm** |
| TDS | n | mean | sd | se | med | min | max | range | 99%CIL | 99%CIU | 0.5%ile | 5%ile | 10%ile | 25%ile | 50%ile | 75%ile | 90%ile | 95%ile | 99.5%ile | TDS |

**Table S1n.** Summary data of mandibular left central incisor (LL1 f & m)

| TDS | n | mean | sd | se | med | min | max | range | 99%CIL | 99%CIU | 0.5%ile | 5%ile | 10%ile | 25%ile | 50%ile | 75%ile | 90%ile | 95%ile | 99.5%ile | TDS |
| --- | --- | --- | --- | --- | --- | --- | --- | --- | --- | --- | --- | --- | --- | --- | --- | --- | --- | --- | --- | --- |
| **LL1Af** | **-** | **-** | **-** | **-** | **-** | **-** | **-** | **-** | **-** | **-** | **-** | **-** | **-** | **-** | **-** | **-** | **-** | **-** | **-** | **LL1Af** |
| **LL1Am** | **-** | **-** | **-** | **-** | **-** | **-** | **-** | **-** | **-** | **-** | **-** | **-** | **-** | **-** | **-** | **-** | **-** | **-** | **-** | **LL1Am** |
| **LL1Bf** | **-** | **-** | **-** | **-** | **-** | **-** | **-** | **-** | **-** | **-** | **-** | **-** | **-** | **-** | **-** | **-** | **-** | **-** | **-** | **LL1Bf** |
| **LL1Bm** | **-** | **-** | **-** | **-** | **-** | **-** | **-** | **-** | **-** | **-** | **-** | **-** | **-** | **-** | **-** | **-** | **-** | **-** | **-** | **LL1Bm** |
| **LL1Cf** | **-** | **-** | **-** | **-** | **-** | **-** | **-** | **-** | **-** | **-** | **-** | **-** | **-** | **-** | **-** | **-** | **-** | **-** | **-** | **LL1Cf** |
| **LL1Cm** | **-** | **-** | **-** | **-** | **-** | **-** | **-** | **-** | **-** | **-** | **-** | **-** | **-** | **-** | **-** | **-** | **-** | **-** | **-** | **LL1Cm** |
| **LL1Df** | 87 | 3.23 | 0.80 | 0.09 | 3.01 | 2.15 | 6.18 | 4.03 | 3.01 | 3.45 | 2.16 | 2.26 | 2.33 | 2.67 | 3.01 | 3.73 | 4.31 | 4.71 | 5.65 | **LL1Df** |
| **LL1Dm** | 94 | 3.44 | 0.80 | 0.08 | 3.28 | 2.28 | 5.17 | 2.89 | 3.23 | 3.66 | 2.31 | 2.44 | 2.54 | 2.72 | 3.28 | 4.04 | 4.67 | 4.76 | 5.07 | **LL1Dm** |
| **LL1Ef** | 105 | 4.67 | 0.85 | 0.08 | 4.71 | 2.77 | 6.15 | 3.38 | 4.45 | 4.88 | 2.84 | 3.15 | 3.57 | 3.97 | 4.71 | 5.30 | 5.71 | 5.92 | 6.13 | **LL1Ef** |
| **LL1Em** | 100 | 4.94 | 0.83 | 0.08 | 5.10 | 3.24 | 6.75 | 3.50 | 4.72 | 5.15 | 3.24 | 3.47 | 3.66 | 4.39 | 5.10 | 5.45 | 5.93 | 6.15 | 6.67 | **LL1Em** |
| **LL1Ff** | 111 | 6.06 | 0.77 | 0.07 | 5.90 | 4.78 | 8.09 | 3.31 | 5.87 | 6.25 | 4.82 | 5.00 | 5.10 | 5.55 | 5.90 | 6.48 | 7.14 | 7.66 | 8.03 | **LL1Ff** |
| **LL1Fm** | 101 | 6.10 | 0.69 | 0.07 | 5.98 | 4.42 | 8.18 | 3.76 | 5.92 | 6.27 | 4.69 | 5.17 | 5.33 | 5.62 | 5.98 | 6.49 | 6.96 | 7.24 | 8.16 | **LL1Fm** |
| **LL1Gf** | 59 | 7.73 | 0.98 | 0.13 | 7.56 | 5.42 | 10.37 | 4.95 | 7.40 | 8.06 | 5.56 | 6.44 | 6.75 | 7.15 | 7.56 | 8.28 | 9.26 | 9.59 | 10.20 | **LL1Gf** |
| **LL1Gm** | 71 | 8.07 | 0.94 | 0.11 | 7.88 | 6.27 | 10.34 | 4.07 | 7.79 | 8.36 | 6.30 | 6.73 | 7.06 | 7.35 | 7.88 | 8.85 | 9.29 | 9.66 | 10.19 | **LL1Gm** |
| **LL1Hf** | 71 | 8.57 | 0.62 | 0.07 | 8.56 | 7.21 | 9.59 | 2.37 | 8.38 | 8.76 | 7.26 | 7.43 | 7.79 | 8.15 | 8.56 | 9.16 | 9.35 | 9.48 | 9.58 | **LL1Hf** |
| **LL1Hm** | 100 | 9.18 | 0.96 | 0.10 | 9.32 | 6.48 | 10.51 | 4.02 | 8.93 | 9.42 | 6.55 | 7.59 | 7.83 | 8.45 | 9.32 | 10.00 | 10.26 | 10.46 | 10.49 | **LL1Hm** |
| TDS | n | mean | sd | se | med | min | max | range | 99%CIL | 99%CIU | 0.5%ile | 5%ile | 10%ile | 25%ile | 50%ile | 75%ile | 90%ile | 95%ile | 99.5%ile | TDS |

**Table S1p.** Summary data of maxillary left third molar (UL8 f & m)

| TDS | n | mean | sd | se | med | min | max | range | 99%CIL | 99%CIU | 0.5%ile | 5%ile | 10%ile | 25%ile | 50%ile | 75%ile | 90%ile | 95%ile | 99.5%ile | TDS |
| --- | --- | --- | --- | --- | --- | --- | --- | --- | --- | --- | --- | --- | --- | --- | --- | --- | --- | --- | --- | --- |
| **UL8Af** | 23 | 10.26 | 1.21 | 0.25 | 10.01 | 7.79 | 12.67 | 4.87 | 9.61 | 10.91 | 7.90 | 8.82 | 9.16 | 9.45 | 10.01 | 10.86 | 11.93 | 12.57 | 12.66 | **UL8Af** |
| **UL8Am** | 28 | 9.82 | 0.90 | 0.17 | 9.86 | 7.96 | 11.44 | 3.48 | 9.38 | 10.26 | 7.99 | 8.38 | 8.75 | 9.10 | 9.86 | 10.51 | 10.91 | 11.12 | 11.40 | **UL8Am** |
| **UL8Bf** | 37 | 10.77 | 1.06 | 0.17 | 10.43 | 9.05 | 13.13 | 4.08 | 10.32 | 11.23 | 9.13 | 9.51 | 9.64 | 9.91 | 10.43 | 11.43 | 12.23 | 12.66 | 13.12 | **UL8Bf** |
| **UL8Bm** | 41 | 10.73 | 0.98 | 0.15 | 10.79 | 9.09 | 13.46 | 4.37 | 10.33 | 11.12 | 9.12 | 9.22 | 9.60 | 10.07 | 10.79 | 11.32 | 11.83 | 12.16 | 13.30 | **UL8Bm** |
| **UL8Cf** | 43 | 11.92 | 1.18 | 0.18 | 11.90 | 9.70 | 14.81 | 5.12 | 11.46 | 12.38 | 9.76 | 10.43 | 10.58 | 10.89 | 11.90 | 12.69 | 13.18 | 13.89 | 14.74 | **UL8Cf** |
| **UL8Cm** | 37 | 12.11 | 1.39 | 0.23 | 11.97 | 9.69 | 16.11 | 6.42 | 11.52 | 12.70 | 9.78 | 10.41 | 10.72 | 11.20 | 11.97 | 12.71 | 13.40 | 15.02 | 16.02 | **UL8Cm** |
| **UL8Df** | 152 | 14.34 | 1.69 | 0.14 | 14.17 | 10.92 | 18.54 | 7.62 | 13.99 | 14.69 | 11.31 | 11.82 | 12.30 | 13.06 | 14.17 | 15.52 | 16.94 | 17.35 | 18.35 | **UL8Df** |
| **UL8Dm** | 135 | 14.21 | 1.59 | 0.14 | 14.06 | 10.89 | 18.56 | 7.66 | 13.86 | 14.56 | 11.24 | 11.95 | 12.24 | 13.09 | 14.06 | 15.15 | 16.41 | 16.74 | 18.45 | **UL8Dm** |
| **UL8Ef** | 58 | 16.49 | 1.90 | 0.25 | 16.47 | 13.24 | 20.32 | 7.08 | 15.85 | 17.13 | 13.25 | 13.54 | 13.75 | 14.96 | 16.47 | 17.77 | 18.93 | 20.02 | 20.32 | **UL8Ef** |
| **UL8Em** | 36 | 16.39 | 1.44 | 0.24 | 16.63 | 14.03 | 19.69 | 5.66 | 15.77 | 17.01 | 14.03 | 14.17 | 14.31 | 15.23 | 16.63 | 17.38 | 18.07 | 18.36 | 19.48 | **UL8Em** |
| **UL8Ff** | 47 | 18.31 | 2.00 | 0.29 | 18.07 | 14.98 | 22.88 | 7.90 | 17.56 | 19.06 | 15.00 | 15.23 | 15.84 | 17.03 | 18.07 | 19.54 | 20.97 | 21.73 | 22.63 | **UL8Ff** |
| **UL8Fm** | 40 | 17.72 | 1.77 | 0.28 | 17.62 | 15.22 | 21.97 | 6.75 | 17.00 | 18.44 | 15.26 | 15.50 | 15.65 | 16.23 | 17.62 | 18.58 | 20.27 | 21.01 | 21.95 | **UL8Fm** |
| **UL8Gf** | 58 | 19.96 | 2.25 | 0.29 | 19.97 | 16.30 | 24.65 | 8.35 | 19.20 | 20.72 | 16.35 | 16.76 | 16.87 | 18.17 | 19.97 | 21.76 | 23.06 | 23.58 | 24.47 | **UL8Gf** |
| **UL8Gm** | 92 | 19.34 | 2.08 | 0.22 | 18.86 | 14.86 | 24.24 | 9.39 | 18.78 | 19.90 | 15.40 | 16.54 | 16.88 | 18.11 | 18.86 | 20.72 | 22.14 | 23.71 | 24.16 | **UL8Gm** |
| **UL8Hf** | 121 | 22.29 | 1.96 | 0.18 | 22.66 | 16.21 | 24.88 | 8.66 | 21.83 | 22.75 | 16.89 | 18.69 | 19.77 | 20.78 | 22.66 | 23.88 | 24.46 | 24.67 | 24.84 | **UL8Hf** |
| **UL8Hm** | 174 | 22.20 | 1.86 | 0.14 | 22.54 | 17.63 | 24.87 | 7.24 | 21.84 | 22.56 | 17.66 | 18.77 | 19.47 | 20.92 | 22.54 | 23.76 | 24.55 | 24.68 | 24.83 | **UL8Hm** |
| TDS | n | mean | sd | se | med | min | max | range | 99%CIL | 99%CIU | 0.5%ile | 5%ile | 10%ile | 25%ile | 50%ile | 75%ile | 90%ile | 95%ile | 99.5%ile | TDS |

**Table S1q.** Summary data of mandibular left third molar (LL8 f & m)

*TDS - Tooth Developmental Stage based on Anglo-Canadian Classification system (Demirjian et al 1973)*

*f - females, m – males, n - number, sd - standard deviation, se - standard error, med - median, min - minimum, max – maximum*

*CIL - Lower Confidence Interval, CIU - Upper Confidence Interval, %ile – Percentile*

*- data for tooth formation not available*

| TDS | n | mean | sd | se | med | min | max | range | 99%CIL | 99%CIU | 0.5%ile | 5%ile | 10%ile | 25%ile | 50%ile | 75%ile | 90%ile | 95%ile | 99.5%ile | TDS |
| --- | --- | --- | --- | --- | --- | --- | --- | --- | --- | --- | --- | --- | --- | --- | --- | --- | --- | --- | --- | --- |
| **LL8Af** | 38 | 9.84 | 1.06 | 0.17 | 9.84 | 7.57 | 11.86 | 4.29 | 9.40 | 10.28 | 7.58 | 7.77 | 8.62 | 9.25 | 9.84 | 10.52 | 11.20 | 11.59 | 11.85 | **LL8Af** |
| **LL8Am** | 53 | 9.89 | 1.02 | 0.14 | 9.95 | 6.86 | 12.67 | 5.81 | 9.53 | 10.26 | 7.15 | 8.50 | 8.78 | 9.21 | 9.95 | 10.52 | 11.00 | 11.32 | 12.50 | **LL8Am** |
| **LL8Bf** | 34 | 11.28 | 1.24 | 0.21 | 11.15 | 9.01 | 13.49 | 4.48 | 10.73 | 11.83 | 9.09 | 9.62 | 9.82 | 10.27 | 11.15 | 12.17 | 12.85 | 13.29 | 13.48 | **LL8Bf** |
| **LL8Bm** | 38 | 11.16 | 1.18 | 0.19 | 11.13 | 9.22 | 15.75 | 6.53 | 10.66 | 11.66 | 9.23 | 9.33 | 9.80 | 10.46 | 11.13 | 11.79 | 12.27 | 12.59 | 15.19 | **LL8Bm** |
| **LL8Cf** | 60 | 12.20 | 1.43 | 0.18 | 12.05 | 9.70 | 16.42 | 6.73 | 11.73 | 12.68 | 9.78 | 10.42 | 10.59 | 10.94 | 12.05 | 12.82 | 14.03 | 14.78 | 16.41 | **LL8Cf** |
| **LL8Cm** | 50 | 12.18 | 1.22 | 0.17 | 12.06 | 9.69 | 16.11 | 6.42 | 11.73 | 12.62 | 9.82 | 10.76 | 11.06 | 11.36 | 12.06 | 12.93 | 13.32 | 13.65 | 16.11 | **LL8Cm** |
| **LL8Df** | 139 | 14.44 | 1.70 | 0.14 | 14.16 | 10.92 | 18.54 | 7.62 | 14.07 | 14.81 | 11.27 | 11.85 | 12.40 | 13.24 | 14.16 | 15.46 | 17.16 | 17.51 | 18.50 | **LL8Df** |
| **LL8Dm** | 120 | 14.07 | 1.30 | 0.12 | 13.99 | 11.72 | 17.71 | 6.00 | 13.76 | 14.37 | 11.73 | 12.05 | 12.32 | 13.25 | 13.99 | 14.89 | 15.73 | 16.55 | 17.45 | **LL8Dm** |
| **LL8Ef** | 54 | 16.07 | 1.33 | 0.18 | 16.09 | 13.24 | 18.72 | 5.48 | 15.60 | 16.54 | 13.25 | 13.94 | 14.51 | 14.98 | 16.09 | 16.94 | 17.66 | 18.20 | 18.72 | **LL8Ef** |
| **LL8Em** | 51 | 15.89 | 1.29 | 0.18 | 15.81 | 12.70 | 18.36 | 5.67 | 15.42 | 16.36 | 12.94 | 14.12 | 14.31 | 14.86 | 15.81 | 16.86 | 17.39 | 18.05 | 18.35 | **LL8Em** |
| **LL8Ff** | 43 | 17.41 | 1.36 | 0.21 | 17.25 | 14.75 | 20.30 | 5.55 | 16.88 | 17.95 | 14.82 | 15.19 | 15.58 | 16.56 | 17.25 | 18.20 | 19.46 | 19.51 | 20.19 | **LL8Ff** |
| **LL8Fm** | 48 | 17.27 | 1.19 | 0.17 | 17.39 | 14.86 | 19.54 | 4.68 | 16.82 | 17.71 | 14.94 | 15.45 | 15.66 | 16.23 | 17.39 | 18.09 | 18.80 | 19.18 | 19.52 | **LL8Fm** |
| **LL8Gf** | 103 | 20.12 | 2.42 | 0.24 | 20.08 | 14.89 | 24.98 | 10.08 | 19.51 | 20.74 | 15.02 | 16.57 | 17.10 | 18.31 | 20.08 | 21.90 | 23.78 | 24.10 | 24.81 | **LL8Gf** |
| **LL8Gm** | 154 | 19.97 | 1.98 | 0.16 | 19.88 | 15.66 | 24.56 | 8.90 | 19.56 | 20.38 | 15.95 | 16.87 | 17.58 | 18.48 | 19.88 | 21.51 | 22.69 | 23.11 | 24.50 | **LL8Gm** |
| **LL8Hf** | 130 | 22.65 | 1.93 | 0.17 | 22.89 | 17.60 | 26.38 | 8.79 | 22.21 | 23.08 | 17.69 | 18.94 | 20.07 | 21.48 | 22.89 | 24.26 | 24.76 | 24.98 | 26.38 | **LL8Hf** |
| **LL8Hm** | 155 | 22.48 | 1.89 | 0.15 | 22.95 | 17.73 | 25.37 | 7.64 | 22.08 | 22.87 | 18.03 | 18.76 | 19.64 | 21.20 | 22.95 | 24.03 | 24.65 | 24.76 | 25.02 | **LL8Hm** |
| TDS | n | mean | sd | se | med | min | max | range | 99%CIL | 99%CIU | 0.5%ile | 5%ile | 10%ile | 25%ile | 50%ile | 75%ile | 90%ile | 95%ile | 99.5%ile | TDS |
